# PI3K/mTOR inhibition induces tumour microenvironment remodelling and sensitises pS6^high^ uterine leiomyosarcoma to PD‐1 blockade

**DOI:** 10.1002/ctm2.1655

**Published:** 2024-05-06

**Authors:** Wout De Wispelaere, Daniela Annibali, Sandra Tuyaerts, Julie Messiaen, Asier Antoranz, Gautam Shankar, Nikolina Dubroja, Alejandro Herreros‐Pomares, Regina E. M. Baiden‐Amissah, Marie‐Pauline Orban, Marcello Delfini, Emanuele Berardi, Thomas Van Brussel, Rogier Schepers, Gino Philips, Bram Boeckx, Maria Francesca Baietti, Luigi Congedo, Kiave Yune HoWangYin, Emilie Bayon, Anne‐Sophie Van Rompuy, Eleonora Leucci, Sebastien P. Tabruyn, Francesca Bosisio, Massimiliano Mazzone, Diether Lambrechts, Frédéric Amant

**Affiliations:** ^1^ Department of Oncology Laboratory of Gynecological Oncology University of Leuven Leuven Belgium; ^2^ Department of Human Genetics Laboratory for Translational Genetics University of Leuven Leuven Belgium; ^3^ Laboratory for Translational Genetics Center for Cancer Biology (CCB) Flemish Institute of Biotechnology (VIB) Leuven Belgium; ^4^ Department of Gynecological Oncology Antoni Van Leeuwenhoek – Netherlands Cancer Institute Amsterdam The Netherlands; ^5^ Department of Medical Oncology Laboratory of Medical and Molecular Oncology (LMMO) Vrije Universiteit Brussel – UZ Brussel Brussels Belgium; ^6^ Department of Imaging and Pathology Translational Cell and Tissue Research University of Leuven Leuven Belgium; ^7^ Department of Pediatrics University Hospitals Leuven Leuven Belgium; ^8^ Department of Biotechnology Universitat Politècnica de Valencia Valencia Spain; ^9^ Laboratory of Tumor Inflammation and Angiogenesis Center for Cancer Biology (CCB) Flemish Institute of Biotechnology (VIB) Leuven Belgium; ^10^ Department of Oncology Laboratory of Tumor Inflammation and Angiogenesis Center for Cancer Biology (CCB) University of Leuven Leuven Belgium; ^11^ Department of Development and Regeneration Laboratory of Tissue Engineering University of Leuven Kortrijk Belgium; ^12^ TRACE, Department of Oncology University of Leuven Leuven Belgium; ^13^ TransCure bioServices Archamps France; ^14^ Department of Pathology University Hospitals Leuven Leuven Belgium; ^15^ Department of Obstetrics and Gynecology University Hospitals Leuven Leuven Belgium

**Keywords:** anti‐PD‐1 therapy, humanized patient‐derived xenograft models, immune‐modulation, PI3K/mTOR inhibitors, resistance, uterine leiomyosarcoma

## Abstract

**Background:**

Uterine leiomyosarcomas (uLMS) are aggressive tumours with poor prognosis and limited treatment options. Although immune checkpoint blockade (ICB) has proven effective in some ‘challenging‐to‐treat’ cancers, clinical trials showed that uLMS do not respond to ICB. Emerging evidence suggests that aberrant PI3K/mTOR signalling can drive resistance to ICB. We therefore explored the relevance of the PI3K/mTOR pathway for ICB treatment in uLMS and explored pharmacological inhibition of this pathway to sensitise these tumours to ICB.

**Methods:**

We performed an integrated multiomics analysis based on TCGA data to explore the correlation between PI3K/mTOR dysregulation and immune infiltration in 101 LMS. We assessed response to PI3K/mTOR inhibitors in immunodeficient and humanized uLMS patient‐derived xenografts (PDXs) by evaluating tumour microenvironment modulation using multiplex immunofluorescence. We explored response to single‐agent and a combination of PI3K/mTOR inhibitors with PD‐1 blockade in humanized uLMS PDXs. We mapped intratumoural dynamics using single‐cell RNA/TCR sequencing of serially collected biopsies.

**Results:**

PI3K/mTOR over‐activation (pS6^high^) associated with lymphocyte depletion and wound healing immune landscapes in (u)LMS, suggesting it contributes to immune evasion. In contrast, PI3K/mTOR inhibition induced profound tumour microenvironment remodelling in an ICB‐resistant humanized uLMS PDX model, fostering adaptive anti‐tumour immune responses. Indeed, PI3K/mTOR inhibition induced macrophage repolarisation towards an anti‐tumourigenic phenotype and increased antigen presentation on dendritic and tumour cells, but also promoted infiltration of PD‐1+ T cells displaying an exhausted phenotype. When combined with anti‐PD‐1, PI3K/mTOR inhibition led to partial or complete tumour responses, whereas no response to single‐agent anti‐PD‐1 was observed. Combination therapy reinvigorated exhausted T cells and induced clonal hyper‐expansion of a cytotoxic CD8+ T‐cell population supported by a CD4+ T_h_1 niche.

**Conclusions:**

Our findings indicate that aberrant PI3K/mTOR pathway activation contributes to immune escape in uLMS and provides a rationale for combining PI3K/mTOR inhibition with ICB for the treatment of this patient population.

## Graphical abstract

Uterine leiomyosarcomas are immunologically cold tumours, rendering them unresponsive to immune checkpoint blockade. We found that aberrant PI3K/mTOR signalling fosters an immunosuppressive tumour microenvironment and contributes to immune checkpoint blockade resistance in uterine leiomyosarcoma. We show that pharmacological inhibition of the PI3K/mTOR pathway induces profound tumour microenvironment remodelling and is able to sensitise these primary resistant tumours to immune checkpoint blockade.

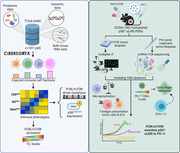

## 1 INTRODUCTION

Uterine leiomyosarcoma (uLMS), while rare, poses a formidable clinical challenge due to its aggressive clinical trajectory and lack of efficacious treatment options.^1^, ^2^ Current therapeutic strategies for localised uLMS entail complete hysterectomy, but tumours frequently recur (53% to 71%), attributed to the proclivity of uLMS for early hematological dissemination.^3^ In the context of metastatic or recurrent disease, anthracycline‐based chemotherapeutic regimens serve as the standard of care. Nevertheless, their efficacy is limited, yielding overall response rates of 25−38%, a median PFS spanning 4.4 to 6.7 months, and an overall survival (OS) less than two years, underscoring the urgent need for new therapeutic options.^1^, ^2^, ^3^

The recent success of immune checkpoint blockade (ICB)‐based therapies in some ‘challenging‐to‐treat cancers’ raises the question whether such therapies could be applicable in uLMS.^4^ Unfortunately, recent phase II clinical trials (NCT02428192, NCT02301039) have shown that uLMS exhibit primary resistance to ICB‐based therapies, including single‐agent anti‐PD‐1 or in combination with anti‐CTLA‐4, with no objective responses among the enrolled patients.^1^, ^5^ A major contributing factor to this primary resistance is the lack of T‐cell infiltration in uLMS, categorising it as a so‐called ‘cold’ tumour. This characteristic has been proposed as the primary reason for its poor responsiveness to ICB.^1^, ^5^ Recent insights into T‐cell exclusion mechanisms have led to the development of combination treatments that can transform ‘cold’ into ‘hot’ tumours, promoting an adaptive anti‐tumour immune response and enhancing their susceptibility to ICB.^6^

Increasing evidence suggests that tumour‐intrinsic signalling pathways can play a crucial role in regulating the anti‐tumour immune response and that targeting these pathways can impact not only cancer cells, but also host immunity.^4^, ^7^ In this context, the PI3K/mTOR pathway has emerged as a central regulator of the tumour microenvironment (TME) and recent investigations have unveiled its potential contribution to ICB‐resistance in some epithelial tumour types, such as breast and colorectal cancer.^8^ *^‐^* ^11^ Aberrant PI3K/mTOR activation occurs in approximately one‐third of uLMS cases, where it correlates with heightened recurrence rates, histological aggressiveness and shorter progression‐free survival (PFS).^12^ Given the frequent dysregulation of this pathway in uLMS, we hypothesised it may be a key contributor to immune evasion and intrinsic ICB‐resistance in this tumour type and that targeting this pathway could enhance response to ICB.

Through an in silico integrated multiomics analysis of 101 LMS‐TCGA patient samples, we identify that over‐activation of the PI3K/mTOR pathway fosters an immunosuppressive TME in (u)LMS. Using a CD34+ humanized PDX model representative of a metastatic pS6^high^ uLMS, we demonstrate that pharmacological inhibition of the PI3K/mTOR pathway elicits anti‐tumour immune responses and sensitises these tumours to PD‐1 blockade. To pinpoint the drivers of response/resistance to ICB in uLMS, we conducted single‐cell transcriptome (scRNA‐seq) and T‐cell receptor repertoire (scTCR‐seq) profiling on serially collected pre‐ and posttreatment tumour biopsies. Our study provides unprecedented insights into the remodelling of the TME of human tumours exposed to PI3K/mTORi and/or PD‐1 blockade within the context of a multilineage human immune system, and a rationale for combining PI3K/mTOR inhibitors and PD‐1 blockade as a promising therapy for treating of pS6^high^ uLMS.

## 2 METHODS

### 2.1 Establishment of PDX models

PDX models were previously established and characterised at the TRACE Platform (UZ/KU Leuven). Tumour tissue fragments from PDX tumours were implanted interscapularly in female NMRI nude mice (minimum 6 weeks old) (Taconic) or NOD‐Prkdc^em26Cd52^Il2rg^emCd22^ mice engrafted with human CD34+ hematopoietic stem cells (HSCs) from three donors (C‐AFP, 760 and C‐AED) with different human leukocyte antigen (HLA)‐A2 serotypes (minimum 24 weeks old) (TransCure bioServices).

### 2.2 Treatment experiments

Mice with tumour volumes of ∼200–300 mm^3^ were divided across treatment arms and treated for up to 30 days. BYL719 (Selleckchem, S2814) was prepared in 5% DMSO (Sigma, 200‐664‐3) + 40% PEG300 (Merck, 25322‐68‐3) + 5% Tween80 (Merck, P4780‐500ML) + 50% ddH_2_O, according to manufacturer instructions. INK128 (Selleckchem, MLN0128) was resuspended in DMSO (Sigma, 200‐664‐3). Both components were diluted in .9% saline and administered daily via oral gavage (INK128, .3 mg/kg + BYL719, 25 mg/kg). Nivolumab (anti‐PD‐1) was provided by Bristol‐Myers Squibb (10 mg/mL solution for infusion), diluted in .9% saline and administered by intraperitoneal (i.p.) injection 2 times per week (10 mg/kg). Tumours were measured 3 times per week with a calipre and volume was calculated using the formula: length × width^2^ × π/6. Mice were euthanised after 30 days of treatment or before, whenever tumours reached a volume of 2000 mm^3^, or in case of any intolerable discomfort. Tumours were harvested and processed into formalin‐fixed, paraffin‐embedded (FFPE) blocks for immunohistochemistry (IHC) or multiplex immunofluorescence (mIF) stainings.

### 2.3 Construction of tissue microarray

Tissue microarray (TMA) was constructed by extracting 2 mm cores from FFPE tumour samples collected from CD34+ HSC humanized EMC041 PDXs at sacrifice, using a TMA grandmaster (3DHISTECH). We selected *n* = 4 tumour blocks from different mice in each treatment condition and extracted between 3 and 4 cores (depending on the size of the tumour) from different tumour regions of each block to construct the TMA.

### 2.4 Multiple iterative labelling by antibody neodeposition (MILAN)

Multiplex immunohistochemistry was performed following a previously established protocol.^13^ Tissue sections (3 μm thick) were obtained from FFPE tumour samples acquired from humanized EMC041 PDXs postsacrifice. Tissue sections underwent dewaxing through sequential immersion in xylene and ethanol baths. Subsequently, antigen retrieval was carried out in Tris‐buffer (pH 8, 10 mM EDTA). Stainings were executed using the Bond RX Fully Automated Stainer (Leica). Details regarding the primary and secondary antibodies utilised are provided in supplementary Table S1. Slides were scanned using the Zeiss Axio Scan Z.1 (Zeiss) at a 10× magnification (.65 μm/pixel). Upon completion of scanning, antibodies were stripped using a buffer containing 1% SDS and β‐mercaptoethanol at 56°C during 30 min. Following stripping, slides were incubated in a washing buffer with periodic changes for 45 min. Each marker was stained separately, by sequentially repeating this staining process.

### 2.5 MILAN quality control and analysis

The quality of the stainings was visually assessed by a digital image expert (JM). At the image level, assessment included cross‐cycle image registration and tissue integrity evaluation. Regions with poor registration or significant tissue deformation and artefacts were excluded. Antibodies exhibiting staining patterns of low confidence upon visual assessment were omitted from the analyses. Image analysis followed a custom pipeline as previously described.^14^ Initially, flat field correction was executed as previously described by Kask and colleagues.^15^ Subsequently, stitching of neighbouring tiles was performed minimising the Frobenius distance of conjunctional regions. To align images from consecutive rounds, transformation matrices were computed using the DAPI channel and applied to the remaining channels.^16^ Postregistration, the accuracy of overlapping was assessed visually. Tissue autoflorescence was then subtracted using the baseline image containing only secondary antibody. Cell segmentation was performed on the DAPI channel employing STARDIST.^17^

### 2.6 MILAN phenotypic identification

Mean fluorescence intensity (MFI) values were normalised to Z‐scores following the recommendation of Caicedo et al.^18^ To mitigate the impact of potential outliers on downstream analyses, Z‐scores were constrained within the [0, 5] range. Single cells were then assigned to known phenotypes through three clustering methods: PhenoGraph,^19^ FlowSom,^20^ and KMeans. Clustering of cells was performed utilising 12 phenotypic markers (ASMA, Desmin, CD14, CD16, CD68, CD86, CD163, CD206, CD3, CD4, CD8, FOXP3). Each cluster was then assigned known cell phenotypes through manual annotation by experts (FMB, JM, double blinded).

### 2.7 Single‐cell RNA/TCR sequencing

Single‐cell RNA and TCR sequencing were conducted to profile transcriptional changes in pre‐ (*n* = 12) and posttreatment (*n* = 3 PD‐1i, *n* = 4 PI3K/mTORi, *n* = 4 PI3K/mTORi+PD‐1i) tumour samples collected from CD34+ HSC humanized PDXs. Tumour biopsies were obtained using 2 mm diameter punch needles (Kai Medical, A01020302) and processed into single‐cell suspensions. The samples underwent mechanical and enzymatic dissociation (2 mg/mL Collagenase P, Sigma Aldrich and .2 mg/mL DNAse I, Roche). Single‐cell TCR sequencing and 5′ gene expression profiling were performed using the Chromium Single Cell V(D)J Solution from 10x Genomics (8000 viable cells/sample). Cell‐barcoded libraries were sequenced on an Illumina NovaSeq6000 system and reads were aligned to the GRCh38 human reference genome using the Cell Ranger Single‐cell software suite.

### 2.8 Single‐cell gene expression analysis

Raw gene expression matrices were generated per sample using CellRanger, imported in R and analysed using Seurat v5 (v4.9.9.9042).^21^ Cell‐level filtering was performed by removing all cells expressing < 200 or > 6000 genes, as well as cells containing < 300 unique molecular identifiers (UMIs), > 15% mitochondrial counts and a log_10_(UMIs)/log_10_(genes) < .8. Gene level filtering was performed by removing all genes expressed in < 10 cells. R package DoubletFinder_v3 (v2.0.3) was used to remove doublets.^22^ Samples were normalised using the *SCTansform* function of Seurat and integrated using the R package Harmony (v0.1.1).^23^ Since every cell has a unique barcode, single‐cell RNA sequencing data could be linked with the single‐cell TCR sequencing data.

### 2.9 Single‐cell RNA sequencing clustering in cell types

For cell clustering, Seurat's default parameters were used unless mentioned otherwise. Principal component analysis (PCA) was performed after adjusting for UMIs, mitochondrial genes, and cell cycle scores (calculated with the *CellCycleScoring* function in Seurat). Only the 1:20 most informative principal components were used to perform dimensional reduction (Uniform Manifold Approximation and Projection (UMAP)). Canonical marker genes were used to annotate cell clusters as known biological cell types.

### 2.10 Trajectory interference analyses

Computationally imputed pseudotime trajectories of the CD8+ T cells were calculated using the R package Slingshot (v2.8).^24^ Due to their distinctive transcriptional profile, the CD8+ T_prolif_ cluster was excluded from the CD8+ T cells. The UMAP matrix as defined with the Seurat R package was passed into Slingshot, considering the CD8+ T_n_ cells as the root state.

### 2.11 Differential expression and pathway analysis

DEG identification was performed using the Seurat functions *FindMarkers* and *FindAllMarkers* (MAST test). A threshold of |LogFC| > .25 and minimum fraction of cells > 3 was applied in all DEG testing. The R package gprofiler2 (v0.2.1) and gene ontology (GO) gene sets were used for gene set enrichment analysis (GSEA) on DEGs. GO (‘C5’) gene sets were used from MSigDB (v2023.1.Hs) and were exported using the R package GSEAbase (v1.62.0). Only significant genes (adjusted *p* < .05) and genes with an average |LogFC| > .25 were used. To perform and visualise GSEA across individual cells, the R package escape (v1.10.0) was used.^25^

### 2.12 TCR repertoire analysis

We recovered 4200 T cells with a TCR sequence. Only productive TCRs were considered. Up to 2999 of the 4200 (71.4%) TCRs detected could be linked to previously annotated T cells by scRNA‐seq. Only TCR clonotypes with the same complementarity‐determining region 3 (CDR3) nucleotide sequences were defined as TCRs. T cells were considered clonal if > 2 cells with the same TCR sequence were found. Cells were further assigned to ‘clonotype bins’ based on TCR frequency using the R package scRepertoire (v1.7.2) with the following thresholds: 0 < *X* < 2 (single), 2 ≤ *X* < 5 (small), 5 < *X* < 20 (medium), 20 < *X* < 100 (large), 100 < *X* < 500 (hyperexpanded).^26^ The R package STARTRAC (v0.1.0) was used to calculate STARTRAC expansion (TCR clonality), Gini index (measure of equality of T‐cell clonotype distribution) and transition index (clonotype sharing between T‐cell subtypes).^27^

### 2.13 CIBERSORTx analysis of TCGA‐SARC dataset

Transcriptomic, genomic, proteomic and clinical data from 101 LMS samples from The Cancer Genome Atlas (SARC‐TCGA, GDAC Firehose Legacy) was accessed using the R package TCGAbiolinks. The downloaded proteomic data consisted of level 3 processed reverse‐phase protein array (RPPA) data as defined on the MD Anderson website (https://bioinformatics.mdanderson.org/public‐software/tcpa/). Based on available bulk RNA‐seq data, the relative composition of the immune component for each sample was estimated using the CIBERSORTx online tool (https://cibersortx.stanford.edu/) with the LM22 immune signature collection.^28^ Based on the composition of their immune cell infiltrate, samples were clustered (*k*‐means clustering) in three distinct immune phenotypes using the R package ComplexHeatmap (v2.13.1). DGE between clusters was performed with the R package DESeq2 (v1.40.1) using default parameters. The R package gprofiler2 (v0.2.1) and gene ontology (GO) gene sets were used for GSEA on DEGs. GO (‘C5’) gene sets were used from MSigDB (v2023.1.Hs) and were exported using the R package GSEAbase (v1.62.0). Only significant genes (adjusted *p* < .05) and genes with a |Log2FC| > .25 were used. Geneset enrichment scores for the pan‐tumour inflamed T‐cell gene signature^29^ were calculated using the R package GSVA (v1.51.0).^30^

### 2.14 Peripheral blood mononuclear cell (PBMC) isolation for T‐cell and human monocyte‐derived macrophage isolation

Buffy coats of 40 mL from three different healthy donors were received from Rode Kruis Vlaanderen and diluted 1:3 in PBS, 1 mM EDTA. The diluted blood was layered over 30 mL Lymphoprep (07811, STEMCELL Technologies) and centrifuged at 1200 × *g* for 20 min at room temperature (RT). The mononuclear interphase was then collected and washed twice with cold PBS‐EDTA. For isolation of human monocyte‐derived macrophages (hMDMS), PBMCs were resuspended in cold buffer (PBS, 2 mM EDTA, .5% BSA) and isolated using CD14‐coated microbeads (130‐050‐201, Miltenyi Biotec) according to manufacturer instructions. Isolated hMDMs were seeded in RPMI supplemented with 10% FBS, 2 mM glutamine, 25 ng/mL human M‐CSF at a concentration of .5 × 10^6^ cells/mL in 6‐well plates and supplemented with additional medium and cytokines after 3 days. Human CD8+ T cells were isolated using the MojoSort™ Human CD8+ T‐cell Isolation Kit (Biolegend, 480129) according to manufacturer instructions. After isolation, cells were seeded in T‐cell medium (RPMI, 10% FBS, 1% pen/strep, 1% MEM nonessential amino acids, 25 μm β‐mercaptoethanol (Gibco) and 1 mM sodium pyruvate (Gibco)). Cells were activated for 48 h with CD3/CD28 Dynabeads (Thermo Fisher Scientific) at a 1:1 bead‐to‐cell ratio. After beads removal, T cells were seeded at a concentration of .5 × 10^6^ cells/mL, and the medium was supplemented with 20 ng/mL human IL‐2.

### 2.15 T‐cell cytokine production

At day 5 after activation, T cells were counted and seeded in 24‐well plates at 1 × 10^6^ cells/mL density and treated for 24 h with INK128 (13 nM) and/or BYL719 (3 μM). Cells were then collected and stimulated for 4 h (37°C) with phorbol 12‐myristate 13‐acetate/ionomycin cell stimulation cocktail (eBioscience, 1:500) in the presence of brefeldin A (Biolegend; 1:1000) and monensin (eBioscience; 1:1000).

### 2.16 Macrophage polarisation

To evaluate the effect of both inhibitors on the polarisation of hMDMs, cells were treated with INK128 (13 nM) and/or BYL719 (3 μM) diluted in RPMI complete at day 7 post isolation, for 24 h. Subsequently, cells were detached using PBS‐EDTA and processed for flow cytometry analysis.

### 2.17 Flow cytometry

Single‐cell suspensions were washed with FACS buffer (PBS, 5% FBS, 2 mM EDTA) and resuspended in Fc block for 15 min. hMDMs were stained with the following antibodies during 45 min at 4°C: Fixable Viability Due eFluor 506 (eBioscience, 65‐0866‐18); anti‐CD14 (BD Pharmingen, 555397); anti‐CD11b (eBioscience, 48‐0112‐82); anti‐CD206(BD Biosciences, 740309); anti‐CD163 (BioLegend, 333622); anti‐HLA‐DR (Thermo Fisher Scientific, 17‐9956‐42); anti‐CD80 (BD Bioscience, 557227); anti‐CD115 (Sony Biotechnology, RT2336540).

CD8+ T cells were stained with the following surface markers: Fixable Viability efluor780 (Thermo Fisher Scientific, 65‐0866‐18) and anti‐CD8 (BioLegend, 980908). Subsequently, cells were incubated for 30 min with Fix/Perm buffer (eBioscience, 00−5523). Cells were washed with permeabilisation buffer (eBioscience, 00−5523) and stained ON (4°C) in permeabilisation buffer with anti‐IFNg (BioLegend, 502542); anti‐TNFa (BioLegend, 986802); anti‐IL‐2 (BioLegend, 500328) and anti‐GZMB (BioLegend, 515406). Cells were subsequently washed and resuspended in FACS buffer. FACS data were acquired using a FACS Fortessa or FACS Symphony (BD Biosciences) and data were analysed using the FlowJo (TreeStar) program.

## 3 RESULTS

### 3.1 PI3K/mTOR pathway over‐activation correlates with an immunosuppressive TME in (u)LMS

**FIGURE 1:**
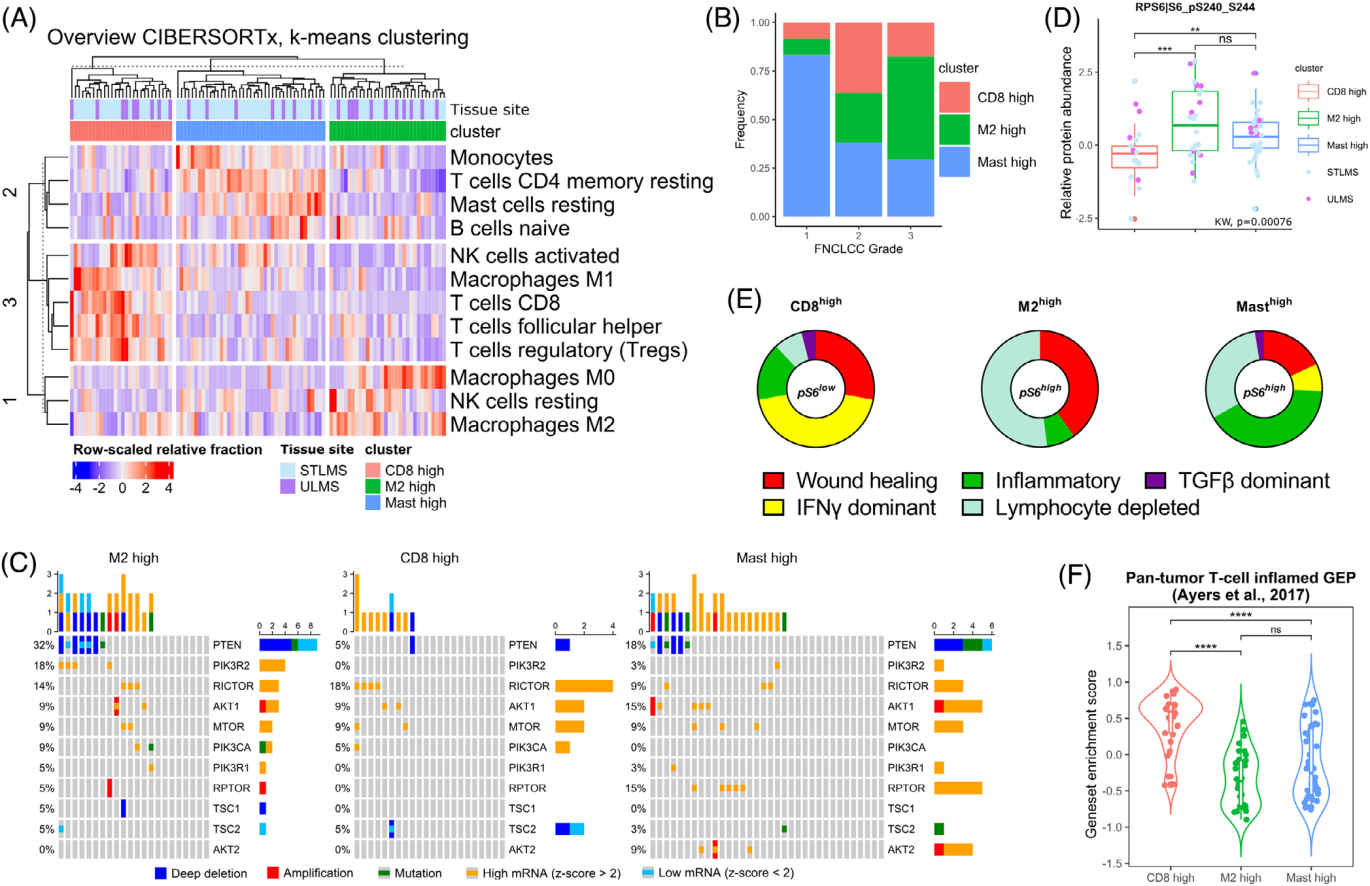
PI3K/mTOR pathway over‐activation is associated with an immune suppressive phenotype in leiomyosarcoma patients. (A) Heatmap showing relative abundance of tumour‐infiltrating immune cell populations in 101 LMS patient samples as determined by deconvolution of bulk RNA expression data from TCGA (TCGA‐SARC, firehose) with CIBERSORTx. Samples have been clustered in three distinct immune phenotypes with *k*‐means clustering. (B) Proportion of samples derived from FNCLCC‐grade 1, 2 or 3 tumours across immune phenotypes. (C) Recurrent mutations, homologous deletions, amplifications and up‐ or downregulation (> 2SD above mean and < 2SD below mean, respectively) of PI3K/mTOR‐driver genes in the three identified immune phenotypes. (D) Levels of phosphorylated (S240/244) S6 protein for all samples stratifying for immune phenotype. *p* Values were calculated using Kruskall–Wallis with Dunn's correction, followed by Wilcoxon rank‐sum test and corrected using Bonferroni for pairwise comparisons. Significant differences are reported as * < .05, ** < .01, *** < .001, **** < .0001. (E) Immune landscaping analysis of the samples performed using the R package R∖ImmuneSubtypeClassifier. (F) Violin plots showing expression of pan‐tumour T‐cell inflamed geneset across samples, stratifying for immune phenotype. *p* Values were calculated using Kruskall–Wallis with Dunn's correction, followed by Wilcoxon rank‐sum test and corrected using Bonferroni for pairwise comparisons. Significant differences are reported as * < .05, ** < .01, *** < .001, **** < .0001.

To investigate whether PI3K/mTOR pathway over‐activation associates with TME immunomodulation in (u)LMS, we performed in silico integrated genomic, transcriptomic and proteomic characterisation of the TCGA cohort of 101 LMS patient samples, consisting of 28 uLMS and 73 nongynecological LMS (STLMS).^31^ Clinical data of included patient samples is represented in supplementary Figure 1A. First, we used the deconvolution tool CIBERSORTx to estimate the abundance of 22 different tumour‐infiltrating immune cell populations in all cases (Figures S1B, 1A, and S1A and E).^32^ Based on the imputed cell fractions of the most discriminative immune populations, we were able to identify three distinct phenotypes characterised by elevated CD8+ T‐cell infiltration (CD8^high^), high mast cell infiltration (mast^high^) or a high ratio of M2/M1‐like polarised macrophages (M2^high^) (Figures 1A and S1C). Samples in the CD8^high^ cluster had significantly elevated levels of CD8+ T cells, regulatory T cells, follicular helper T cells, activated natural killer (NK) cells and anti‐tumourigenic M1‐like macrophages, compared to the other phenotypes. The M2^high^ and CD8^high^ clusters had comparable levels of M2‐like macrophage infiltration, but the increased presence of M1‐like macrophages in the CD8^high^ cluster resulted in a significantly higher M1/M2 ratio, compared to the M2^high^ cluster (*p*‐value = 9.05e‐7). The mast^high^ cluster was characterised by high levels of mast cells, low levels of CD8+ T cells and was enriched for low‐grade tumours (Fédération Nationale des Centres de Lutte Contre le Cancer (FNCLCC)‐grade 1), while the CD8^high^ and M2^high^ groups had higher relative abundances of high‐grade tumours (FNCLCC‐grade 2 and 3) (Figures 1B and S1F). Across immune phenotypes, no significant differences were noted in sample site of origin.

Uterine and nongynecological LMS harbour frequent alterations in driver genes associated with the PI3K/mTOR pathway.^31^ The in silico analysis showed that 8/28 (28%) of samples in the CD8^high^ cluster carry PI3K/mTOR‐driver gene alterations, compared to 20/32 (62.5%) in the M2^high^ and 30/41 (73%) in the mast^high^ clusters (Figure 1C). Notably, compared to the CD8^high^ group, both the M2^high^ and mast^high^ clusters were also enriched for PTEN deletions, mutations or downregulation (*p*‐value = .0366), which has been shown to drive constitutive PI3K/mTOR pathway activation.^33^ *^,^* ^34^

For 82/101 (81%) LMS samples analysed, reverse‐phase protein array (RPPA) data were available. We assessed the abundance of S240/244 phosphorylated RPS6, reflecting PI3K/mTOR pathway over‐activation, in the CD8^high^, M2^high^ and mast^high^ clusters and found that the CD8^high^ cluster displayed significantly lower levels of S6 phosphorylation compared to the M2^high^ and mast^high^ clusters (Figure 1D), while no significant differences in the abundance of total RPS6 protein were found (Figure S1G). This suggests that LMS with high levels of PI3K/mTOR pathway activation display low tumour T‐cell infiltration and increased infiltration of M2‐like polarised macrophages or mast cells.

To further investigate the role of the PI3K/mTOR pathway in shaping the TME in (u)LMS, we pursued tumour immune‐landscape mapping according to the six pan‐cancer immune landscapes identified by Torsson et al.^35^ We found that the pS6^low^ CD8^high^ cluster was strongly enriched for the IFNγ‐dominant immune‐landscape, while the pS6^high^ M2^high^ cluster was almost exclusively enriched for the lymphocyte depleted and wound healing immune landscapes. The pS6^high^ mast^high^ cluster was likewise enriched for the lymphocyte depleted, but also for the inflammatory immune‐landscape (Figure 1E). IFNγ‐dominant immune landscapes are associated with high levels of CD8+ T‐cell and M1‐like polarised macrophage infiltration, reflecting a dominant type I immune response.^35^ Lymphocyte depletion and wound healing immune landscapes accentuate tumoural macrophage infiltrates with primarily immunosuppressive M2‐like orientation, consistent with an immunosuppressed TME and poor prognosis.^35^ *^,^* ^36^

Increasing evidence indicates that tumour‐infiltrating immune cells determine immunotherapy response in different sarcoma subtypes.^37^ *^,^* ^38^ To assess whether the PI3K/mTOR pathway controlled immune‐landscape in uLMS could influence immunotherapy response, we evaluated the expression of a pan‐tumour T‐cell inflamed gene signature, which reliably predicts clinical response to PD‐1 blockade in 10 different cancer types, in our cohort of (u)LMS‐TCGA samples.^29^ We found this gene set to be significantly higher expressed in the pS6^low^ CD8^high^ compared to the pS6^high^ M2^high^ and Mast^high^ clusters (Figure 1F). Collectively, these data suggest that PI3K/mTOR pathway over‐activation is associated with an immunosuppressive TME in LMS, potentially contributing to ICB‐resistance.

### 3.2 PI3K/mTOR inhibitors promote tumour T‐cell infiltration in pS6^high^ uLMS PDXs

**FIGURE 2:**
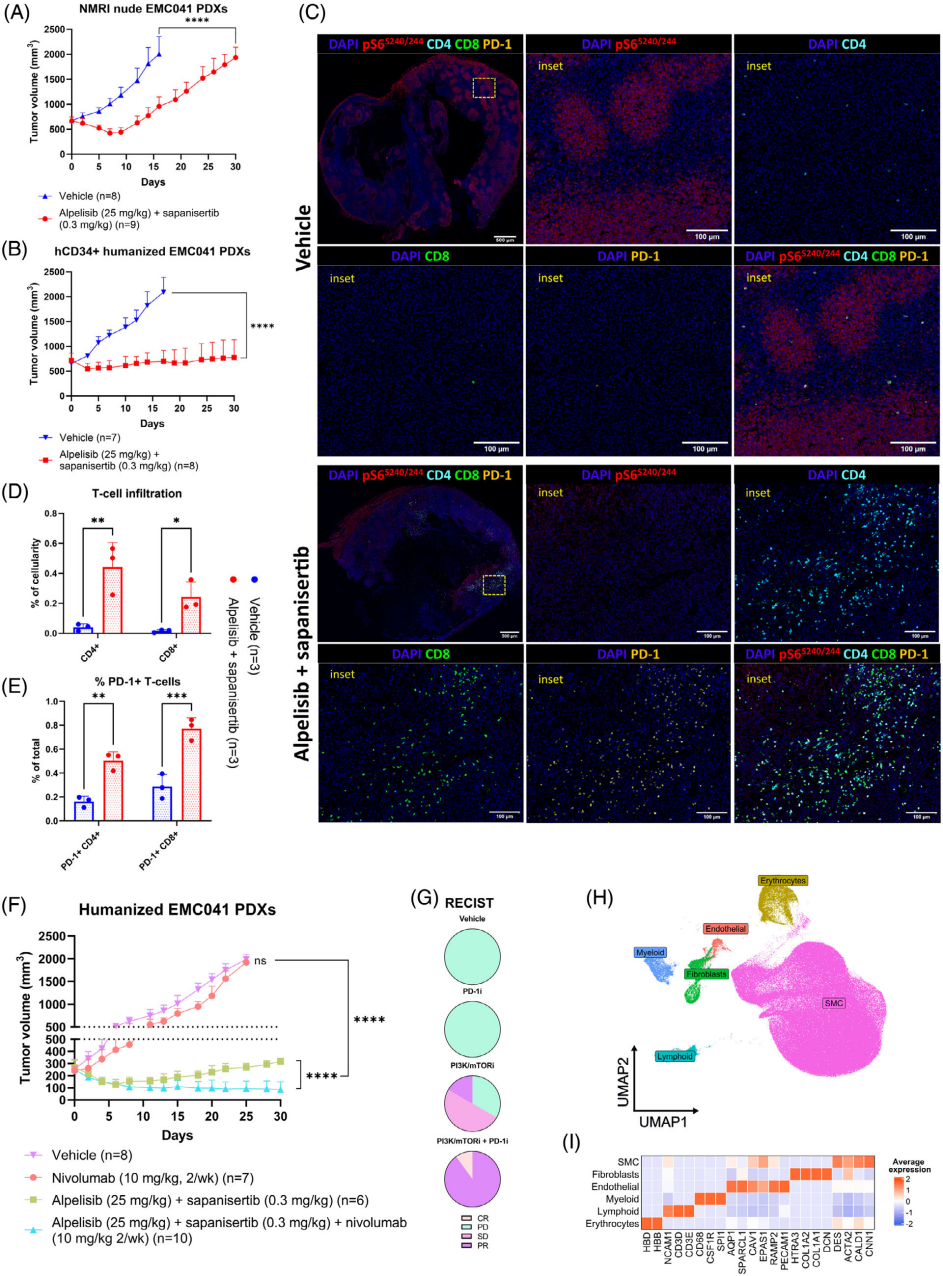
PI3K/mTOR inhibitors promote tumour T‐cell infiltration and sensitise pS6^high^ uLMS CD34+ humanized PDX model to anti‐PD‐1 therapy. (A) Immunocompromised mice (NMRI nude) were engrafted with a patient‐derived uLMS lung metastasis (EMC041) and treated with (i) sapanisertib (.3 mg/kg/day) + alpelisib (25 mg/kg/day) or (ii) vehicle. Tumour volume was measured three times per week with a calipre. (B) CD34+ humanized mice were engrafted with EMC041 and treated with (i) sapanisertib (.3 mg/kg/day) + alpelisib (25 mg/kg/day) or (ii) vehicle. Tumour volume was measured three times per week with a calipre. (C) Multiplex immunofluorescence (mIF) analysis of representative sections of C34+ humanized EMC041 PDX tumours after treatment. (D) Quantification of CD4+ and CD8+ T‐cell infiltration in sections of vehicle‐ and PI3K/mTORi‐treated CD34+ humanized EMC041 PDX tumours (*n* = 3 tumours per condition) (expressed as % of total cellularity). (E) Quantification of PD‐1 expression on CD4+ and CD8+ T cells in sections of vehicle‐ and PI3K/mTORi‐treated CD34+ humanized EMC041 PDX tumours (*n* = 3 tumours per condition) (expressed as % of total CD8+ or CD4+ T cells). (F) CD34+ HSC humanized mice engrafted with EMC041 were treated with (i) sapanisertib (.3 mg/kg/day) + alpelisib (25 mg/kg/day), (ii) nivolumab (10 mg/kg, 2/wk), (iii) sapanisertib (.3 mg/kg/day) + alpelisib (25 mg/kg/day) + nivolumab (10 mg/kg, 2/wk) or (iv) placebo. Tumour volume was measured three times per week with a calipre. (G) Summary of responses according to RECIST per treatment arm. (H) Tumour punch biopsies were collected from the humanized EMC041 PDXs (pre‐ and posttreatment in all treatment arms) and subjected to single‐cell RNA/TCR sequencing. UMAP of cells colour coded for indicated cell types. (I) Heatmap showing the expression of conserved marker genes used to identify cell populations. For all experiments data points and error bars represent mean values and SEM. *p* Values were calculated using ANOVA and two‐sample *t*‐tests and are reported as ns > .05, * < .05, ** < .01, *** < .001, **** < .0001. The number of mice per treatment arm and number of tumours analysed by mIF are indicated in the figures for each experiment.

To evaluate how the dual PI3K/mTOR inhibition would affect immune responses in uLMS, we implanted a pS6^high^ uLMS lung metastasis PDX model (EMC041), previously established at our lab,^12^ in both athymic nude mice (immunocompromised mice lacking T cells) and CD34+ hematopoietic stem cell (HSC) humanized mice (NOD‐Prkdc^em26Cd52^Il2rg^emCd22^). We then treated the tumour‐bearing mice with the combination of a PI3Kα‐selective inhibitor (alpelisib) and mTORC1/2 inhibitor (sapanisertib). In nude mice tumours initially responded, but quickly developed resistance and started to exponentially grow seven days after treatment initiation (Figure 2A). By contrast, tumours implanted in humanized mice showed prolonged growth suppression in response to PI3K/mTORi (up to 30 days). This suggests the presence of a reconstituted immune system can, at least in part, contribute to tumour growth control during treatment (Figure 2B). Although implantations in the different backgrounds did not occur simultaneously and could not be systematically compared, growth rates of vehicle‐treated tumours were consistent between backgrounds. In the nude and humanized mice tumours reached the predefined threshold of 2000 mm^3^ (exclusion criteria) 16 ± 1 and 17 ± 2 days after inclusion, respectively. This further supports our hypothesis that the different outcome of PI3K/mTORi treatment in humanized versus nude mice could be related to an immune‐potentiating effect of these inhibitors.

Multiplex‐IF analysis in the humanized mice showed that PI3K/mTORi‐treated tumours displayed higher CD4+ and CD8+ T‐cell infiltration in the tumour bed and lower pS6^S240/244^ levels (Figure 2C and D), compared to vehicle‐treated tumours. This suggests that pharmacological inhibition of the PI3K/mTOR pathway may enhance T‐cell infiltration, which could underlie the observed responses. In addition, these tumour‐infiltrating T cells expressed high levels of PD‐1 (Figure 2C and E), which has been associated with response to anti‐PD‐1 therapy.^39^

### 3.3 PI3K/mTOR inhibitors prime a pS6^high^ uLMS hCD34+ humanized PDX model for PD‐1 blockade

To test whether the increased infiltration of PD‐1+ T cells upon dual PI3K/mTOR inhibition could sensitise tumours to ICB‐based therapy in vivo, we treated CD34+ humanized EMC041 PDXs with: (i) nivolumab (anti‐PD‐1), (ii) alpelisib + sapanisertib, (iii) nivolumab + alpelisib + sapanisertib or (iv) vehicle. Single‐agent PD‐1 inhibition did not induce any degree of tumour growth control, suggesting the lung metastasis lesion from which the PDX was established to be intrinsically resistant to anti‐PD1 therapy. When treated with alpelisib + sapanisertib, tumour growth suppression was observed, leading to a mixture of partial responses (PR) (1/6), stable (SD) (3/6) or progressive disease (PD) (2/6). Importantly, combination therapy of nivolumab and alpelisib + sapanisertib, further inhibited tumour growth, leading to PR (9/10) and one complete response (CR) (1/10) (Figure 2F and G). Multiplex immunofluorescence staining for pS6^S240/244^ confirmed successful attenuation of PI3K/mTOR signalling in tumours treated with PI3K/mTOR or PI3K/mTOR + PD‐1 inhibitors (Figure S2C and D).

To dissect drivers of response, we performed scRNA‐seq on tumour biopsies collected at baseline (pretreatment, d1) and at the time of sacrifice (posttreatment, d30 or whenever tumours reached a volume of 2000 mm^2^) from *n* = 14 CD34+ humanized EMC041 PDXs (*n* = 3 vehicle, *n* = 3 PD‐1i, *n* = 4 PI3K/mTORi, *n* = 4 PI3K/mTORi+PD‐1i). We obtained high‐quality sequencing data for 248 930 cells, in which we detected an average of 3091 genes/cell. Subsequent analysis identified several clusters, which based on marker gene expression were identified as malignant smooth muscle cells (SMC), fibroblasts, endothelial cells, erythrocytes, lymphoid and myeloid cells (Figure 2H and I). No apparent batch effects for individual tumours or treatment condition were observed (Figure S2E). In line with aforementioned findings, when comparing the relative cell fractions (% of total) across treatment conditions, we observed a significant enrichment of the lymphoid fraction in the PI3K/mTORi‐ (Wilcoxon, *p*‐value = .0414) and PI3K/mTORi+PD‐1i‐treated (Wilcoxon, *p*‐value = .0013) versus pretreatment condition (Figure S2F), confirming that PI3K/mTORi are able to promote infiltration of T cells in pS6^high^ uLMS.

### 3.4 Tumours treated with PI3K/mTOR inhibitors and PD‐1 blockade display increased effector versus exhausted CD8+ T‐cell ratios and enhanced CD4+ T_h_1 functionality

**FIGURE 3:**
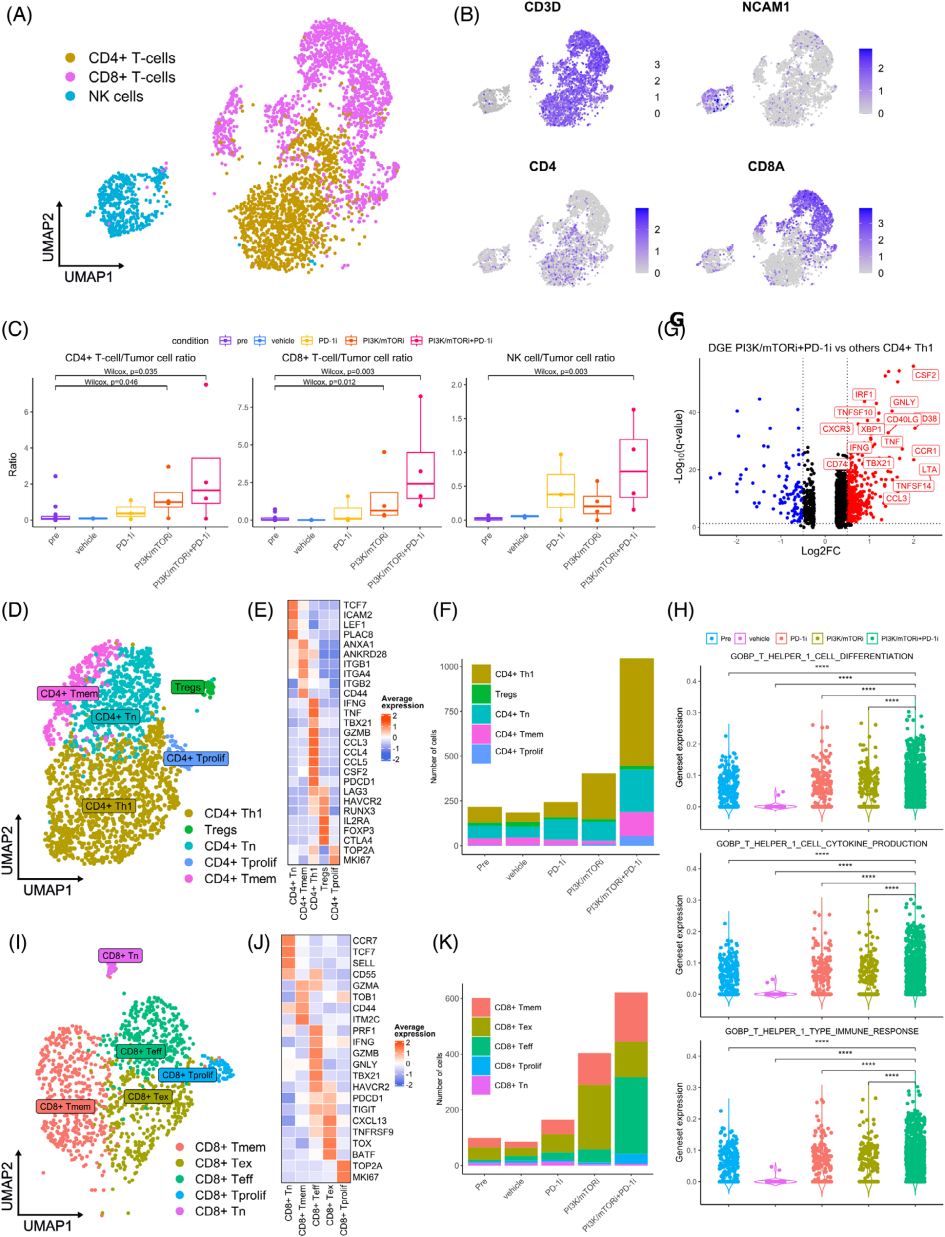
Combination therapy of PI3K/mTOR inhibitors and PD‐1 blockade increases T‐cell infiltration and ratio of effector vs exhausted CD8+ T cells. (A) Lymphoid cells were sub clustered into T/NK cells based on expression of marker genes (CD3E, CD4, IL7R, CD8, NCAM1). (B) UMAP showing the scaled expression of marker genes used to identify T/NK cells. (C) Ratio of CD4+ and CD8+ T cells and NK cells to tumour cells in each treatment condition. *p* Values were calculated using Kruskal–Wallis with Dunn's correction, followed by Wilcoxon rank‐sum test and corrected using Bonferroni for pairwise comparisons. Significant differences are reported as * < .05, ** < .01, *** < .001, **** < .0001. (D) CD4+ T cells were sub clustered into 5 phenotypes. Based on the expression of marker genes, we identified naïve (CD4+ T_n_), memory (CD4+ Tmem), Th1 (CD4+ Th1), regulatory (CD4+ Tregs) and proliferating (CD4+ T_prolif_) T cells. UMAP is colour coded for the indicated cell phenotypes. (E) Heatmap showing the scaled expression of marker genes used to identify CD4+ T‐cell phenotypes. (F) Relative contribution of each CD4+ T‐cell phenotype in the different treatment arms. (G) Volcano plot showing differential gene expression in CD4+ Th1 cells in PI3K/mTORi + PD‐1i versus other treatment conditions. Significantly up‐ and downregulated genes (*q*‐value < .05, |Log2FC| > .25) are shown in red and blue, respectively. (H) Violin plots showing gene set enrichment scores for the indicated pathways in the CD4+ T‐cell population. *p* Values were calculated using Kruskal–Wallis with Dunn's correction, followed by Wilcoxon rank‐sum test and corrected using Bonferroni for pairwise comparisons. Significant differences are reported as * < .05, ** < .01, *** < .001, **** < .0001. (I) CD8+ T cells were sub clustered into 5 phenotypes. Based on expression of marker genes, we identified naïve (CD8+ T_n_), memory (CD8+ T_mem_), effector (CD8+ T_eff_), exhausted (CD8+ T_ex_) and proliferating (CD8+ T_prolif_) T cells. UMAP is colour coded for the indicated cell phenotypes. (J) Heatmap showing the scaled expression of marker genes used to identify CD8+ T‐cell phenotypes. (K) Relative contribution of each CD8+ T‐cell phenotype in the different treatment arms.

Since we observed a significant increase in the lymphoid fraction pre‐ versus posttreatment in the PI3K/mTORi‐ and PI3K/mTORi+PD‐1i‐treated tumours, we further analysed the TILs and, based on marker gene expression, identified NK‐cell, CD4+ and CD8+ T‐cell populations (Figure 3A and B). Considering the levels of TILs pre‐ versus posttreatment (normalised against tumour cell population), we observed a significant increase in both the CD4+ and CD8+ T‐cell/tumour cell ratios, in the PI3K/mTORi‐ and PI3K/mTORi+PD‐1i‐treated conditions but not in the PD‐1i‐ or vehicle‐treated tumours (Figure 3C). These results were corroborated through multiple iterative labelling by antibody neodisposition (MILAN)‐analysis of FFPE tissue sections obtained from CD34+ humanized EMC041 PDXs at sacrifice across the various treatment groups. Our analysis revealed a significant increase of CD4+ and CD8+ T‐cell infiltration in tumours treated with PI3K/mTORi and PI3K/mTORi+PD‐1i, as compared to tumours treated with vehicle or PD‐1i alone (Figure S3A and B).

To probe the T‐cell functional states, T cells were subclustered into their established phenotypes based on marker gene expression.^40^ *^‐‐^* ^42^ In the CD4+ T‐cell cluster, we identified subpopulations of naïve (CD4+ T_n_), type‐1 helper (CD4+ T_h_1), memory (CD4+ T_mem_), regulatory (T_regs_) and proliferating (CD4+ T_prolif_) T cells (Figure 3D and E). A strong enrichment of the CD4+ T_h_1 (IFNG^hi^, TNF^hi^, TBX21^hi^, GZMB^hi^) subpopulation was observed exclusively in the PI3K/mTOR+PD‐1i‐treated tumours (Figure 3F) and DGE analysis revealed this population upregulates the expression of immune response promoting factors (IFNG, TNF, CSF2, TNFSF10 (TRAIL), TNFSF14 (LIGHT), CCL3) (Figure 3G). Consistently, gene set enrichment analysis in the CD4+ T‐cell population also showed upregulated expression of gene sets related to T_h_1 differentiation, T_h_1 cytokine production and T_h_1 immune response, suggesting improved CD4+ T_h_1 activity in the PI3K/mTORi+PD‐1i‐treated tumours, not found in other treatment conditions (Figure 3H).

In the CD8+ T‐cell cluster, we identified naïve (CD8+ T_n_), effector (CD8+ T_eff_), exhausted (CD8+ T_ex_), memory (CD8+ T_mem_) and proliferating (CD8+ T_prolif_) subpopulations (Figure 3I). The CD8+ T_eff_ showed high expression of cytotoxicity markers (PRF1, IFNG, GZMB, GNLY), master regulator of type 1 immune responses transcription factor T‐bet (TBX21) and expression of immune checkpoints (PDCD1, HAVCR2, TIGIT). The CD8+ T_ex_ retained high immune checkpoint expression, while displaying lower levels of cytotoxic‐activity genes (PRF1, IFNG, GZMB, GNLY) and upregulated expression of markers associated with T‐cell exhaustion (TOX, BATF, CXCL13, TNFSFR9) (Figure 3J).^42^ *^,^* ^43^ When comparing relative abundance of these subpopulations across treatment conditions, the CD8+ T‐cell population in the PI3K/mTORi condition was enriched for T_ex_ compared to pre‐, vehicle‐ and PD‐1‐treated tumours. By contrast, an enrichment of the T_eff,_ population and depletion of the T_ex_ population was observed in the PI3K/mTORi+PD‐1i‐ versus PI3K/mTORi‐treated tumours (Figure 3K). In addition, we observed an increase in the expression of cytotoxicity (PRF1, GNLY, GZMB) and proliferation (MKI67) markers in the CD8+ T cells in PI3K/mTORi+PD‐1i‐treated tumours (Figure S4A). These data indicate PI3K/mTOR inhibitors promote tumour T‐cell infiltration, but these T cells are marked by a transcriptional profile consistent with exhaustion and loss of effector function (T_ex_). When treated with combined PD‐1i and PI3K/mTORi, an enrichment of a CD8+ T_eff_ population with enhanced cytotoxicity and proliferative capacity was observed, resulting in a favourable T_eff_/T_ex_ ratio. Furthermore, we observed a strong CD4+ T_h_1 enrichment, uniquely in the PI3K/mTORi+PD‐1i‐treated tumours, which suggest the presence of this supportive CD4+ T_h_1 niche is crucial in maintaining the proliferative and cytotoxic functions of CD8+ T_eff_ cells (Figure 3K).^41^

### 3.5 Combination therapy of PI3K/mTOR inhibitors and PD‐1 blockade reverts exhaustion and induces clonal hyper‐expansion of CD8+ T_eff_ population

Since it has been reported that clonally expanded T cells underlie the therapeutic efficacy of ICB, we used scTCR‐seq to define clonotypes based on shared TCR sequences in 4200 T cells.^44^ *^,^* ^45^ We considered T cells with shared sequences in ≥2 cells to be clonally expanded (E).^41^ Conversely, T cells with shared sequences in < 2 cells were considered nonexpanded (NE). Clonally expanded T cells were further assigned to ‘clonotype bins’ based on their TCR frequency (hyperexpanded (500 < *X* > 100), large (100 < *X* > 20), medium (20 < *X* > 5) or small (5 < *X* ≥2)).

**FIGURE 4:**
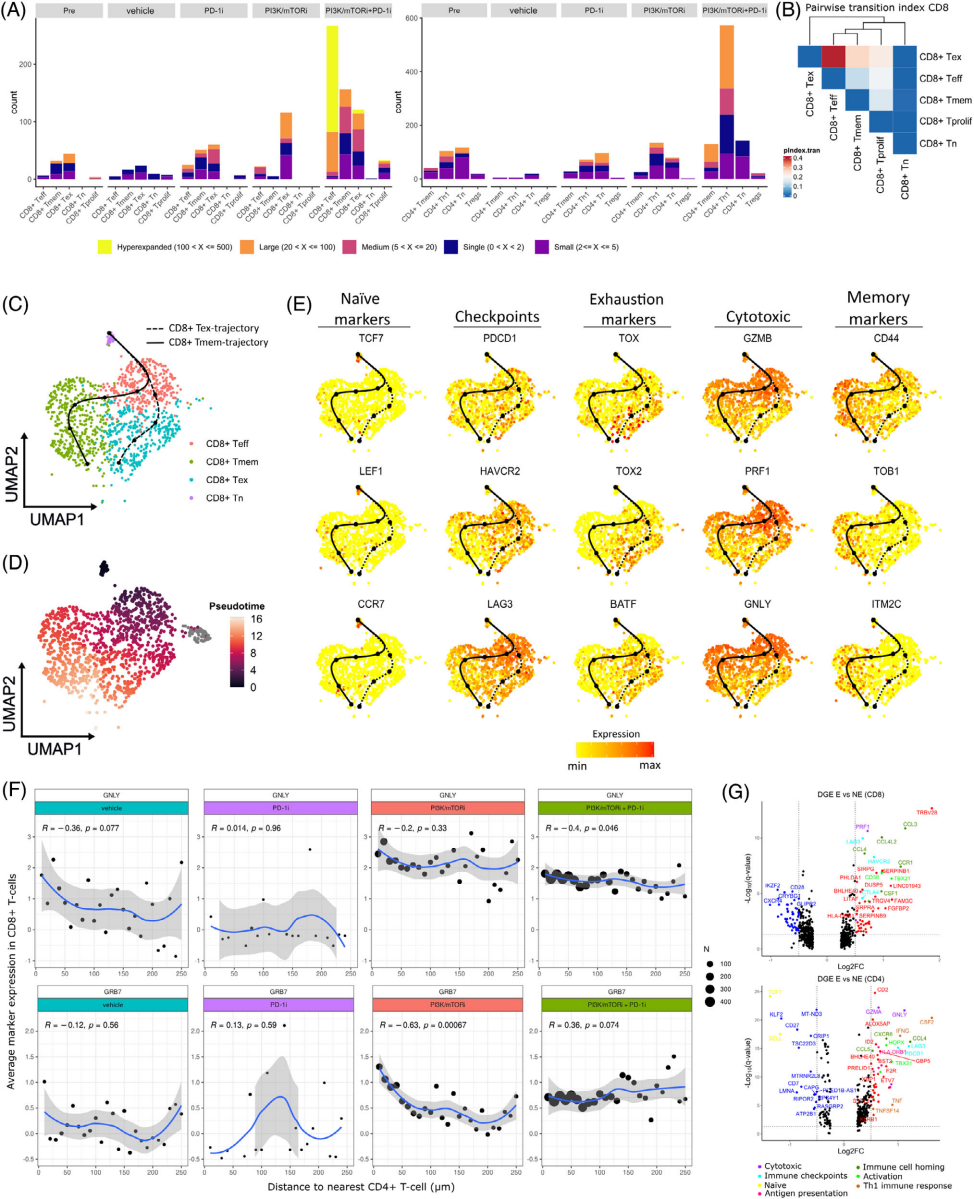
Combination therapy of PI3K/mTOR inhibitors and PD‐1 blockade induces clonal hyper‐expansion of the effector CD8+ T‐cell population and counteracts T‐cell exhaustion. (A) CD8+ and CD4+ T cells were assigned to ‘clonotype bins’ based on their clonotype frequency (hyperexpanded: TCR was found in > 100 T cells, large: > 20 and < 100 T cells, medium: > 5 and < 20, small: > 1 and < 5 or single: > 0 and < 1). Stacked columns show clonotype distribution of CD8+ T‐cell clonotypes per subtype stratifying for treatment condition. (B) Pairwise transition index (measure for clonotype sharing between specific CD8+ T‐cell subtypes) was calculated using the R package STARTRAC between all CD8+ T‐cell subtypes. (C) UMAP colour coded for CD8+ T‐cell phenotypes with pseudotime trajectories for CD8+ T cells based on R∖Slingshot. (D) UMAP of CD8+ T cells colour coded for pseudotime. (E) UMAPs of CD8+ T cells showing expression of marker and functional genes along T_mem_ and T_ex_ trajectories. (F) Average expression of cytotoxicity markers on CD8+ T cells plotted against the distance to the closest CD4+ T cell in micrometers. (G) Volcano plot showing differential gene expression in expanded (E) versus nonexpanded (NE) CD8+ and CD4+ T cells. Significantly up‐ and downregulated genes (*q*‐value < .05, |Log2FC| > .25) are shown in red and blue, respectively and selected genes are colour coded according to functionality.

When comparing clonal T‐cell expansion and Gini index (measure for the inequality of TCR distribution) across treatment conditions, we found these to be significantly higher post‐ versus pretreatment in the PI3K/mTORi‐ and PI3K/mTORi+PD‐1i treatment conditions, while no significant expansion was observed in PD‐1i‐ or vehicle‐treated tumours (Figure S4B). In the PI3K/mTORi‐treated tumours clonal T cells were almost exclusively confined to the CD8+ T_ex_‐cell population, while in the PI3K/mTORi+PD‐1i‐treated tumours, clonally expanded T cells primarily belonged to the CD8+ T_eff‐_ and CD4+ T_h_1‐cell population (Figures 4A and S4C, E and F). To assess dynamic relations between T‐cell subsets, we performed TCR tracking using the STARTRAC R package and found significant sharing of common TCRs (high pairwise transition index) between the CD8+ T_eff_ and T_ex_ clusters, which suggests these clusters are likely developmentally connected (Figures 4B and S4D).^27^ *^,^* ^41^

Next, we generated computationally imputed pseudotime trajectories for the CD8+ T cells using Slingshot.^24^ We considered the CD8+ T_n_ cells as the root state of the trajectories, since this population showed the lowest Gini index, indicating a high diversity of TCR clones. We observed two distinct trajectories: T_n_ cells were connected to T_eff_ cells, branching to form T_mem_ or T_ex_ cells (Figures 4C and D and S4G). Along both trajectories, we observed an upregulation of prominent T‐cell activation marker CD44, distinguishing T_eff_ cells from their naïve counterparts.^46^ As opposed to the T_ex_ trajectory and consistent with previous studies, CD44 expression remained high in T cells along the T_mem_ trajectory and an upregulation of memory marker expression (TOB1, ITM2C) was observed (Figure 4E).^47^ Along the CD8+ T_ex_ trajectory, we observed high expression of immune checkpoints (PDCD1, HAVCR2, LAG3) and a decreased expression of cytotoxic‐activity genes (GZMB, PRF1, GNLY). Consistently, we also observed an increase in the expression of exhaustion markers (TOX, TOX2, BATF), suggesting a progressive loss of effector function as T cells become more exhausted (Figure 4E). T cells on the T_ex_ and T_mem_ trajectory in the PI3K/mTORi+PD‐1i‐treatment condition had lower mean pseudotime scores compared to the other treatment conditions (Figure S3H), suggesting that the enriched T‐cell population in the PI3K/mTORi+PD‐1i‐treated biopsies are characterised by more pronounced effector function and early exhaustion, while tumour‐infiltrated T cells in the PI3K/mTORi‐treated biopsies are more terminally exhausted.

To validate our findings, we examined the expression of activation (CD69, OX40) and exhaustion markers (LAG3, TIM3) markers on T cells in FFPE tissue sections from humanized EMC041 PDX tumours collected at sacrifice. Employing a previously published model,^48^ we classified each CD8+ T cell on a functional spectrum ranging from ‘active’ (CD69^high^ and/or OX40^high^) to ‘exhausted’ (TIM3^high^ and/or LAG3^high^CD69^low^OX40^low^) (Figure S3C and D). Our analysis revealed that tumours treated with PI3K/mTORi and PI3K/mTORi+PD‐1i exhibited significantly higher levels of tumour‐infiltrating activated CD8+ T cells (% of activated CD8+ T cells/mm^2^ of tumour area) compared to those treated with vehicle or PD‐1i alone (Figure S3E). While not reaching statistical significance, there was a clear trend towards elevated levels of exhausted CD8+ T cells in the PI3K/mTORi‐treated tumours compared to those treated with PI3K/mTORi+PD1i. Additionally, we assessed the expression of cytotoxicity markers (GRB7/GZMB and GNLY) on CD8+ T cells and proliferation marker Ki67 on both CD4+ and CD8+ T cells. An enrichment of GRB7+ and GNLY+ CD8+ T cells (cells/mm^2^ tumour area) was observed in tumours treated with PI3K/mTORi and PI3K/mTORi+PD1‐i compared to those treated with vehicle or PD‐1i alone (Figure S3F). Similarly, tumours treated with PI3K/mTORi or PI3K/mTORi+PD‐1i exhibited higher proportions proliferating Ki67+ T cells (% of total CD4+ or CD8+ T cells) (Figure S3G). Neighbourhood analysis revealed that CD8+ T cells in tumours treated with PI3K/mTORi or PI3K/mTORi+PD‐1i were in closer proximity to CD4+ T cells and expressed elevated levels of cytotoxicity markers when adjacent to CD4+ T cells, highlighting the supportive role of CD4+ T cells in maintaining the cytotoxic capabilities of CD8+ T cells (Figure 4E). Collectively, these results confirm that tumours treated with PI3K/mTORi and/or PI3K/mTORi+PD‐1i are enriched for an actively proliferating cytotoxic CD4+ and CD8+ T‐cell population, indicative of an ongoing adaptive anti‐tumour immune response.

Finally, we identified DEGs between expanded and nonexpanded CD4+ and CD8+ T cells (Figure 4G). Expanding CD4+ and CD8+ T cells exhibited high cytotoxicity (PRF1, GZMB, GZMA, GNLY, NKG7, FASLG), activation/differentiation (TBX21, HOPX), immune cell‐homing signals (CCL3/4/5/4L2) and immune checkpoint expression (HAVCR2, LAG3, CTLA4) compared to nonexpanding T cells. In the CD4+ T‐cell compartment, nonexpanded T cells were more naïve (SELL, TCF7, IL7R), while their expanding counterparts exhibited high T_h_1 activity (IFNG, TNF, CSF2, TNFSF14 (LIGHT)) and antigen presentation (HLA‐DRA/DPB1/DPA1/DRB1, CD74).

Together these findings suggest that by combining PI3K/mTORi with PD‐1i in the EMC041 PDX model, we not only promote tumour T‐cell infiltration but also revert T‐cell exhaustion and stimulate the clonal hyper‐expansion of a cytotoxic CD8+ T‐cell population supported by a clonally expanded CD4+ T_h_1 niche.

### 3.6 PI3K/mTOR inhibition correlates with M1‐like macrophage repolarisation and increased expression of dendritic cell antigen cross‐presentation machinery

**FIGURE 5:**
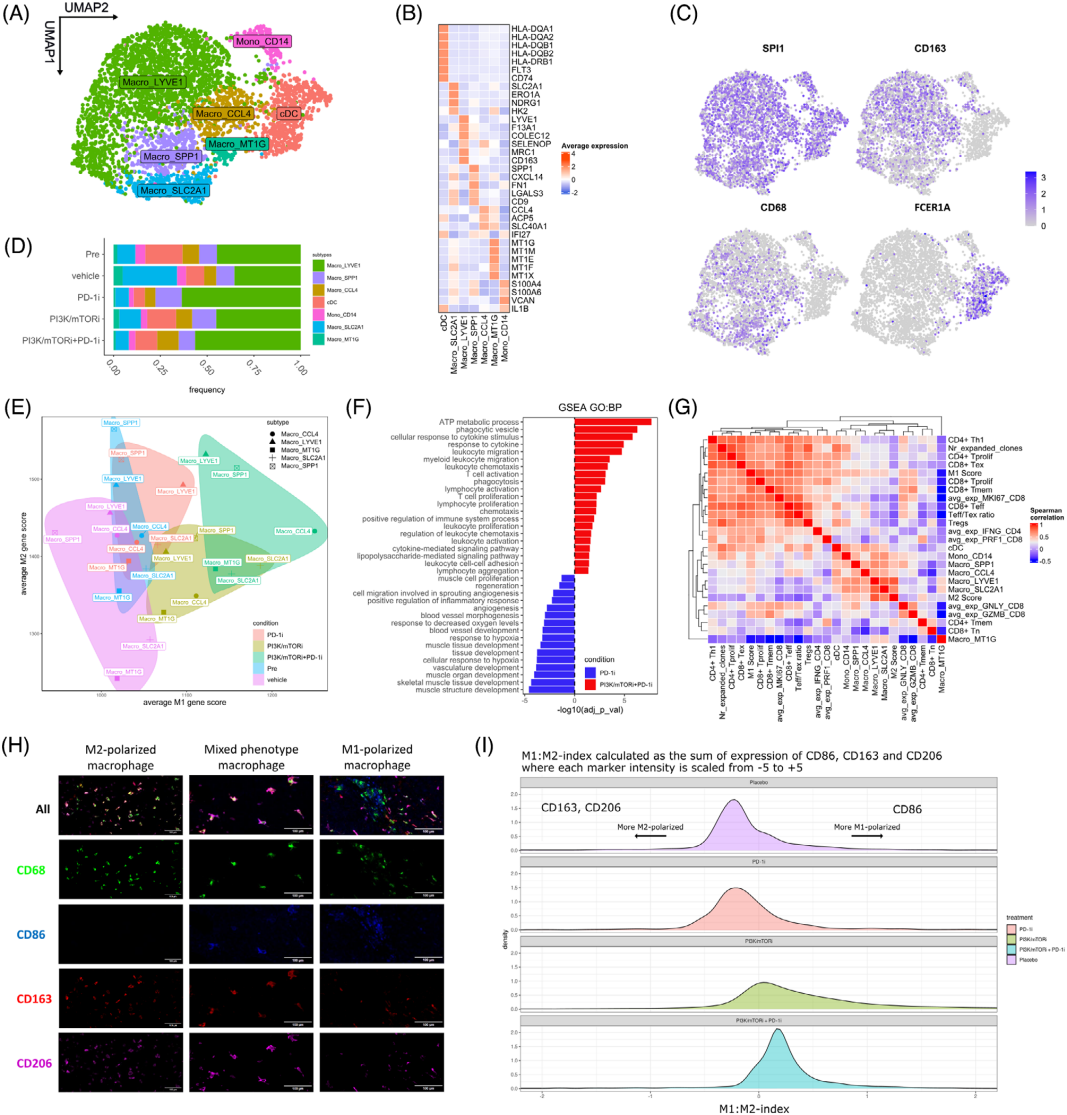
Repolarisation of tumour‐infiltrated macrophages towards an anti‐tumourigenic M1‐like phenotype and enhanced dendritic cell antigen presentation in PI3K/mTOR + PD‐1 inhibitor‐treated tumours. (A) UMAP of myeloid cells sub clustered into 1 monocyte, 5 macrophage and 1 dendritic cell cluster. (B) Heatmap showing scaled expression of marker genes used to identify myeloid subtypes. (C) UMAP of myeloid fraction showing expression of marker genes used to identify monocyte, macrophage and dendritic cell subclusters. (D) Relative contribution of each myeloid subtype (in %) in the different treatment arms. (E) Scatterplot of the mean M2 versus mean M1 score for all the macrophage cell clusters, stratifying for treatment condition. (F) Barplot representing pathways upregulated in the PI3K/mTORi + PD‐1i, shown in red and downregulated (upregulated in PD‐1i) indicated in blue. Only significantly up‐ and downregulated genes (*q*‐value < .05, |Log2FC| > .25) were used to perform gene set enrichment analysis. (G) Spearman correlation analysis with the number of expanded T‐cell clonotypes, relative abundance of cellular phenotypes, average expression of cytotoxicity and proliferation markers in CD8+ T cells, average IFNɣ expression in CD4+ T cells and mean M1 and M2 gene signature scores in the macrophage populations. (H) Representative images of CD68, CD86, CD163 and CD206 staining (MILAN‐method) of TMA constructed from tumour cores of C34+ humanized EMC041 PDX tumours. Macrophages showed M1‐like, M2‐like and mixed expression phenotypes. (I) Assignment of polarisation state for each macrophage across the M1:M2 spectrum using scaled, three marker index. Plot shows the distribution of M1:M2 index values for all macrophages in tumours stratifying for treatment groups.

Given that macrophages and dendritic cells act as important mediators of tumour immunity, we subclustered myeloid cells into a monocyte/macrophage (CD68^hi^, CD163^hi^) and dendritic cell (cDC) (FCER1A^hi^) fractions (Figure 5A and C). Within the monocyte/macrophage fraction, we identified five previously established macrophage phenotypes and one monocyte cluster (Figure 5A and B).^40^ *^,^* ^41^ *^,^* ^49^ No major differences in the composition of the monocyte/macrophage fraction were found between treatment conditions (Figure 5D). We assessed the expression of an ‘alternatively activated’ (M2)‐ or ‘classically activated’ (M1)‐gene signature in the macrophage population.^50^ Macrophages showed expression of both signatures, consistent with previous studies.^51^ However, macrophages in the PI3K/mTORi‐ and PI3K/mTORi+PD‐1i‐treated tumours expressed higher M1‐gene signatures compared to other conditions, suggesting that macrophages in tumours treated with PI3K/mTORi and PI3K/mTORi+PD‐1i adopt an M1‐like anti‐tumourigenic polarisation (Figure 5E). Pathway enrichment analysis based on DEGs revealed that macrophages in PI3K/mTORi‐ and PI3K/mTORi+PD‐1i‐exposed tumours upregulate pathways related to phagocytosis, T‐cell recruitment/activation and LPS‐mediated signalling (M1‐like), while downregulating pathways associated with tissue remodelling and angiogenesis (M2‐like) (Figure 5F). Notably, SPP1+ and LYVE1+ macrophages exhibited higher M2 signatures compared to CCL4+, SLC2A1+ and MTG1+ macrophages, consistent with their pro‐tumourigenic role previously described in literature.^52^ *^,^* ^53^

To validate the finding that macrophages are reeducated towards an M1‐like polarisation in tumours treated with PI3K/mTORi and PI3K/mTORi+PD‐1i, we evaluated the expression of M1 (CD86) and M2 (CD163, CD206) markers on FFPE tissue sections from humanized EMC041 PDX tumours collected at sacrifice, using MILAN‐analysis (Figure 5H). Each macrophage was assigned an M1:M2 index based on joint measurement of CD86, CD163 and CD206 markers (Figure 5I). Tumours treated with PI3K/mTORi or PI3K/mTORi+PD‐1i tended to have higher densities of M1 polarised macrophages as compared to those treated with vehicle or PD‐1i alone, which further indicates macrophages in PI3K/mTORi‐ and PI3K/mTORi+PD‐1i‐treated tumours are polarised towards an M1‐like phenotype.

Similar to macrophages, the numbers of cDCs did not vary between treatment conditions. However, cDCs in the PI3K/mTORi+PD‐1i‐treated tumours upregulated expression of genes related to antigen presentation via major histocompatibility complex class II (MHC II) (HLA‐DRB1, HLA‐DQB1, HLA‐DPB1, HLA‐DQB2, CD74) compared with cDCs in tumours treated with PD‐1i, PI3K/mTORi‐ or vehicle (Figure S5A). Consistently, upregulated pathways in cDCs from PI3K/mTORi+PD‐1i‐treated tumours were associated with antigen processing and presentation via MHC II, and positive regulation of T‐cell activation, suggesting enhanced antigen cross‐presentation, which is essential for T‐cell priming (Figure S5B).

Finally, we explored correlation between infiltration, expansion and functionality of T cells with the presence of myeloid subtypes and macrophage polarisation. T‐cell expansion, relative frequency of CD8+ and CD4+ T cells, T_eff_/T_ex_ ratio and expression of cytotoxicity markers positively correlated with the expression of an M1‐gene signature and relative frequency of cDCs. On the contrary, the expression of an M2‐signature inversely correlated with these parameters (Figure 5G). We did not observe strong positive or negative correlation with the relative frequency of macrophage subtypes, which suggests that macrophage polarisation rather than their abundance may affect T‐cell infiltration, expansion and function in these tumours.

### 3.7 Cancer cells upregulate expression of antigen presentation machinery genes in tumours treated with PI3K/mTOR and PD‐1 inhibitors

**FIGURE 6:**
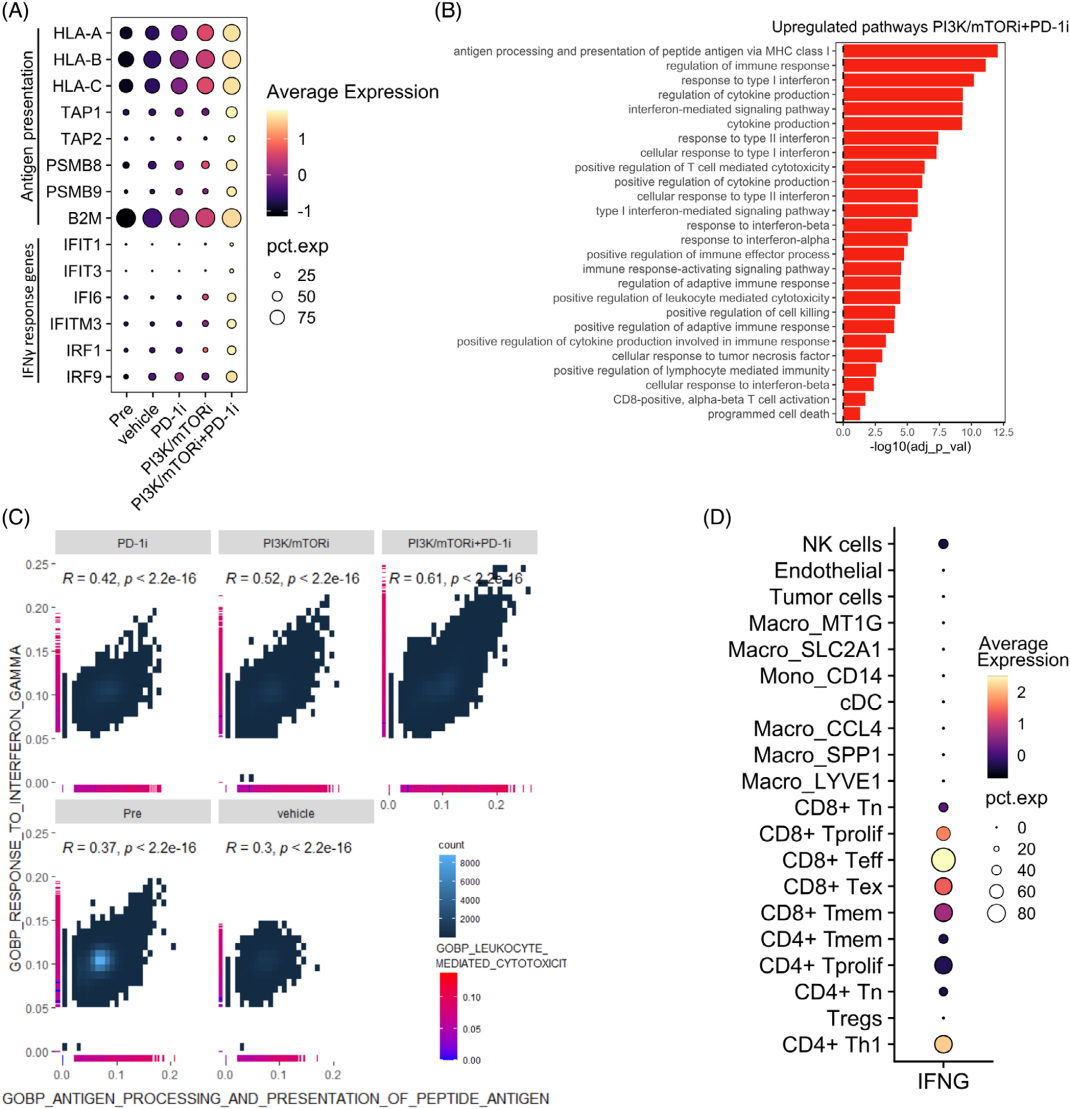
Combination therapy of PI3K/mTOR inhibitors and PD‐1 blockade induces IFNɣ‐mediated upregulation of antigen presentation machinery in tumour cells. (A) Violin plots showing gene expression of IFNγ response genes and antigen processing and presentation genes in the tumour cell population, stratifying for treatment conditon. (B) DGE was performed in the tumour cell population PI3K/mTORi+PD‐1i versus other treatment conditions. Significantly up‐ and downregulated genes were used to perform gene set enrichment analysis. Pathways upregulated in the PI3K/mTORi + PD‐1i‐treated tumours. (D) Scatterplot of gene set enrichment scores in the tumour cells, stratifying for treatment condition (GO:BP RESPONSE TO INTERFERRON GAMMA versus GO:BP ANTIGEN PROCESSING AND PRESENTATION OF PEPTIDE ANTIGEN). For each tumour cell, gene set enrichment score for GO:BP LEUKOCYTE MEDIATED CYTOTOXICITY is indicated at the side of the plot. (E) Dotplot showing expression of IFNɣ in all different cell populations identified from scRNA‐seq of tumour biopsies. The colour of the dots indicates scaled expression and the size of the dots the percentage of IFNɣ expressing cells.

Since T‐cell activity relies on tumour antigen presentation by cancer cells, we further investigated the cancer cell compartment of our tumours. We found antigen presentation and IFNɣ‐response genes upregulated in PI3K/mTORi‐exposed tumours and most prominently in PI3K/mTORi+PD‐1i‐treated tumours, compared to pretreatment, vehicle‐ or PD‐1i‐treated tumours (Figure 6A). Consistently, pathways related to antigen processing, presentation and IFNɣ‐response signalling were upregulated (Figure 6B). We observed a positive correlation in tumour cells between the expression of gene sets related to antigen processing presentation, response to IFNγ and leukocyte‐mediated cytotoxicity, which was strongest in the PI3K/mTORi+PD‐1i‐tumours (Figure 6C). Assessing which cell types produce IFNγ, we found that IFNγ was almost exclusively expressed in the lymphoid population and was highest in the CD4+ T_h_1 and CD8+ T_eff_ cells (Figure 6D). These data suggest that tumour cells in PI3K/mTORi‐ and most prominently PI3K/mTORi+PD‐1i‐treated tumours upregulate antigen processing and presentation in response to IFNγ produced by the enriched tumour‐infiltrating T‐cell population, which in turn makes them more prone to T‐cell attack.

### 3.8 PI3K/mTOR inhibitors do not affect T‐cell function and macrophage polarisation at tumour cell cytotoxic concentrations in vitro

Since the PI3K/mTOR pathway is one of the key signalling pathways controlling activation, functionality and cell fate decisions in T cells and macrophages, we assessed whether treatment with PI3K/mTORi directly interfered with T‐cell function and macrophage polarisation in vitro.^54^ *^,^* ^55^

First, we established a cell line from an EMC041 PDX tumour and determined the half‐maximal growth inhibitory concentration (GI_50_) for alpelisib (2.80 ± 1.21 μM) and sapanisertib (13.28 ± 1.07 nM) (Figure S5C and D). Next, we cultured CD8+ T cells and monocyte‐derived macrophages (MDMs) isolated from healthy human donors in the presence or absence of alpelisib and/or sapanisertib at the GI_50_ for the EMC041 cell line. We assessed the effect of these inhibitors on macrophage polarisation by assessing their expression of M1 (CD80, HLA‐DR) and M2 (CD163, CD206) markers and observed no significant differences after exposure to alpelisib and/or sapanisertib compared to the control condition (Figure S5E). Similarly, production of effector cytokines (IL‐2, IFNγ and TNFα) did not significantly differ in CD8+ T cells exposed to alpelisib and/or sapanisertib compared to the control condition (Figure S5F). These data suggest that alpelisib and sapanisertib (alone or in combination) at tumour cell cytotoxic concentrations, do not directly affect T‐cell effector functions or macrophage polarisation.

## 4 DISCUSSION

Presently, the standard‐of‐care therapeutic options are only effective in a fraction of metastatic or recurrent uLMS patients.^2^ ULMS are characterised by a scarcity of TILs, classifying them as immunologically ‘cold’ tumours and rendering them unresponsive to ICB.^1^ *^,^* ^5^ *^,^* ^56^ Nevertheless, the underlying mechanisms responsible for this observed resistance remain poorly understood. A better understanding of these mechanisms is imperative to pave the way for tailored therapies aimed at overcoming resistance.^57^

Extensive research has documented the role of the PI3K/mTOR pathway in promoting tumour cell intrinsic mechanisms of survival and proliferation. More recently, it has been unveiled that this pathway is also involved in orchestrating immune evasion, particularly by driving T‐cell exclusion.^58^ *^,^* ^59^ This holds significant relevance to uLMS, given the frequent dysregulation of this pathway (1/3 of cases), and some indications that hyper‐activation of the PI3K/mTOR pathway might contribute to resistance to ICB in this specific tumour type.^12^ *^,^* ^34^ *^,^* ^60^ *^–^* ^62^ For instance, George et al. reported a case involving a treatment‐naïve metastatic uLMS patient who experienced complete disease remission, with the exception of one treatment‐resistant metastatic lesion, upon receiving pembrolizumab. Genomic sequencing revealed a loss of function PTEN mutation unique to the resistant lesion, implicating this mutation in immunotherapy evasion.^34^ Furthermore, PIK3CA mutations and increased mTOR signalling have been linked to poor response to ICB in other cancer types, including breast, colorectal and clear cell renal cancer.^63^ *^,^* ^64^ In addition, previous studies have indicated that PI3K/mTOR inhibitors can synergise with PD‐1 blockade in syngeneic mouse models of melanoma, prostate, colorectal and breast cancer.^65^ *^‐‐^* ^68^ However, a comprehensive analysis of the role of the PI3K/mTOR pathway in ICB‐resistance in uLMS and its therapeutic implications is still lacking.

To address these gaps, we conducted an in silico integrated multiomics analysis the TCGA‐SARC dataset to evaluate the role of the PI3K/mTOR pathway in immunomodulation in a large cohort of 101 (u)LMS patient samples. Consistent with findings in other cancer types, our analysis revealed a negative correlation between PI3K/mTOR pathway over‐activation and TIL levels, implying that this pathway plays a role in T‐cell exclusion and immune evasion in (u)LMS.^63^ *^,^* ^64^

To investigate whether pharmacological inhibition of this pathway could be therapeutically exploited to enhance response to ICB, we performed an in‐depth analysis to decipher the dynamic changes within the TME following PI3K/mTOR inhibition and/or PD‐1 blockade, using a clinically relevant CD34+ HSC humanized PDX model of a metastatic uLMS. Our findings revealed that, in line with results from recent clinical trials in uLMS, this tumour model is intrinsically resistant to single‐agent PD‐1 blockade.^1^ *^,^* ^5^ Tumour response did not differ between vehicle‐ and PD‐1i‐treated PDXs. In both the vehicle‐ and PD‐1i‐treated tumours, we observed low levels of TILs, which did not significantly increase from pre‐ to posttreatment, suggesting that the lack of observed response is due to impaired T‐cell infiltration. By contrast, treating the PDXs with PI3K/mTOR inhibitors significantly increased T‐cell infiltration pre‐ versus posttreatment, albeit these T cells became exhausted over time and lost their effector function. The combination of PI3K/mTOR inhibitors with PD‐1 blockade effectively reinvigorated the exhausted T‐cell population, reinstating their effector function and leading to clonal hyper‐expansion of a cytotoxic CD8+ T_eff_ population, ultimately resulting in partial or complete tumour responses. Furthermore, we observed a clonal expansion and enrichment of a CD4+ T_h_1 population exclusively in tumours treated with combination therapy of PI3K/mTOR inhibitors and PD‐1 blockade, implying this T‐cell population is essential in supporting and maintaining CD8+ T‐cell effector function. Additionally, in tumours subjected to PI3K/mTOR inhibitors, alone or in combination with anti‐PD‐1 therapy, we observed the repolarisation of macrophages towards an anti‐tumourigenic (M1‐like) phenotype. This was accompanied by the induction of a transcriptional profile in dendritic cells that suggested enhanced antigen cross‐presentation, as well as IFNγ‐mediated upregulation of genes associated with antigen processing and presentation in tumour cells. By contrast, macrophages in the vehicle‐ and PD‐1i‐treated tumours predominantly displayed an M2‐like orientation, upregulating genes and pathways associated with angiogenesis and tissue remodelling. These findings indicate profound remodelling of the lymphoid and myeloid TME in response to PI3K/mTOR inhibition, which closely aligns with the results of a recent study that examined the effects of genetic and pharmacological inhibition of pik3ca and pik3cb in a genetically engineered mouse model of breast cancer with concurrent loss of Pten and Trp53. In this study, both genetic and pharmacological inactivation of pik3cb and pik3ca similarly resulted in enhanced T‐cell and M1‐like macrophage infiltration, along with improved dendritic cell antigen cross‐presentation.^9^

At present, two PI3K inhibitors with activity against the α‐isoform have garnered FDA approval, namely alpelisib (BYL719) and copanlisib (BAY80‐ 6946). Notably, alpelisib exclusively targets the α‐isoform primarily expressed in tumour cells, while copanlisib exhibits dual specificity for the α‐ and δ‐isoforms, with the latter being primarily expressed in lymphocytes. In our study, we opted for alpelisib (BYL719), due to the hypothesis that the use of a δ‐inhibitor might potentially interfere with anti‐PD‐1 responses by directly impairing T‐cell activity. Dual PI3K/mTOR inhibition has shown superior efficacy compared to targeting PI3K alone in combination with ICB in preclinical mouse models of breast cancer.^69^ *^,^* ^70^ Current FDA‐approved mTOR inhibitors (everolimus, serolimus and tesirolimus) exclusively target mTORC1. However, their efficacy is limited since they attenuate an mTORC1‐dependent negative feedback loop, which causes compensatory over‐activation of mTORC2 leading to therapy resistance.^71^ *^,^* ^72^ Therefore, we combined alpelisib with sapanisertib, an mTORC1/2 inhibitor, which has shown more potent anticancer activity compared to first‐generation mTOR inhibitors and is currently in clinical trials for various indications, including glioblastoma, nonsmall cell lung, bladder and breast cancer (NCT02133183, NCT04250545, NCT03047213, NCT02465060).^72^

Through experiments involving exposure of CD8+ T cells and MDM isolated from healthy human donors to tumour cell cytotoxic concentrations of alpelisib and sapanisertib, alone or in combination, we established that these inhibitors, at these doses, do not exert direct influence on T‐cell function or macrophage polarisation. Although further investigation on the direct effect of PI3K/mTORi on T cells and macrophages is warranted, these data suggest that these inhibitors do not hinder immune responses by directly interfering with the functionality of T cells or macrophages in the tumours and might be suitable candidates for combination therapy with PD‐1 blockade.

The rarity of uLMS, coupled with the limited availability of reliable preclinical models, has curtailed the development of new therapeutic options for this vulnerable patient population. Regrettably, over the past decade prognosis has seen little improvement. Our study, conducted using a highly clinically relevant CD34+ HSC humanized PDX model, provides compelling evidence of immune evasion orchestrated by the PI3K/mTOR pathway. Our study underscores the value of employing humanized mouse models in conjunction with omics profiling as a powerful strategy for identifying novel treatment approaches, particularly in the context of rare cancers. Furthermore, it offers a strong rationale for the clinical development of combination therapies involving PI3K/mTOR inhibitors and immunotherapy in pS6^high^ uLMS, heralding a potentially transformative approach for patients in dire need of effective treatments.

## 5 CONCLUSION

In conclusion, our research provides evidence elucidating the pivotal role of PI3K/mTOR pathway over‐activation in orchestrating T‐cell exclusion and fostering ICB‐resistance in uLMS. Moreover, our findings provide a compelling rationale for the clinical exploration of combination therapies involving PI3K/mTOR inhibition and immunotherapy in pS6^high^ uLMS. This innovative approach holds the potential to revolutionise the management of uLMS by providing them access to immunotherapy.

## AUTHOR CONTRIBUTIONS

Conceptualisation: D.L., F.A., S.T., W.D.W., D.A. Methodology: B.B., D.L., E.L., M.F.B., A.A., F.A., F.M.B., S.T., W.D.W., D.A., M.M., M.D. M.P.O., G.S. Software: B.B., A.A., G.P., W.D.W., G.S. Validation: T.V.B., A.A., W.D.W., G.S. Formal analysis: B.B., A.A., G.P., W.D.W., M.P.O., A.H.‐P., L.C., G.S. Investigation: D.L., E.L., M.F.B., E.B., F.A., J.M., R.B.A., A.V.R., W.D.W., K.H., S.T., M.P.O., A.H.‐P., L.C., A.‐S.V.R., N.D. Resources: B.B., D.L., E.L., M.F.B., F.A., G.P., S.T., W.D.W., K.H., S.T., E.B. Data curation: B.B., G.P., W.D.W. Writing – original draft preparation: F.A., W.D.W. Writing – editing & review: D.L., B.B., E.L., M.F.B., F.A., J.M., G.P., F.M.B., R.B.A., S.T., W.D.W., D.A., A.H.‐P., A.‐S.V.R. Visualisation: A.A., F.A., F.M.B., W.D.W., M.P.O., G.S. Supervision: D.L., F.A., F.M.B., D.A., M.M., M.D., S.T. Project administration: F.A., S.T., D.A. Funding acquisition: D.L., F.A., S.T., W.D.W., D.A.

## CONFLICT OF INTEREST STATEMENT

All authors declare that they have no competing interests.

## FUNDING INFORMATION

Research was funded by Kom op tegen Kanker (Stand up to Cancer), the Flemish Cancer Society grant (#11040). TRACE staff are supported by Stichting Tegen Kanker grant (#2016‐054). W.D.W is supported by an Emmanuel van der Schueren (EvdS) grant. A.H.‐P. was funded by the Horizon Europe Framework Programme under the Marie Skłodowska‐Curie Postdoctoral Fellowship 2021 (#101064216) and a Margarita Salas fellow from the program for the retraining in the Spanish university system of the Universitat Politècnica de València and the Spanish Ministry of Universities funded by the European Union‐Next Generation. M.P.O. was funded by Fonds Wetenschappelijk Onderzoek – Vlaanderen (FWO) (1124423N).

## ETHICS STATEMENT

The establishment and characterisation of patient‐derived xenografts have been approved by the Ethics Committee Research UZ/KU Leuven under signature of informed consent (S54185). In vivo treatment experiments (described in the S67784 research protocol) have been approved by the KU Leuven EC (P159/2018), and performed according to the ARRIVE guidelines. Buffy coats from healthy donors for isolation of PBMCs were acquired from Rode Kruis Vlaanderen under institutional approval RKOV_19015.

## CONSENT FOR PUBLICATION

All authors have read and approved the final version of the manuscript.

## Supporting information

Supporting Information

Supporting Information

Supporting Information

Supporting Information

Supporting Information

Supporting Information

## Data Availability

The transcriptomic, genomic and proteomic data from 101 LMS patients used in this study are available for download from The Cancer Genome Atlas (TCGA). Sequencing data from single‐cell RNA/TCR sequencing of tumour biopsies from humanized PDXs and raw multiplex immunofluorescence images are available upon request at http://biokey.lambrechtslab.org.
