# Supplementary figures and images for: PI3K/mTOR inhibition induces tumour microenvironment remodelling and sensitises pS6high uterine leiomyosarcoma to PD‐1 blockade
Source: Clin Transl Med. 2024 May 6;14(5):e1655. doi: 10.1002/ctm2.1655 (PMC11074386; doi:10.1002/ctm2.1655)

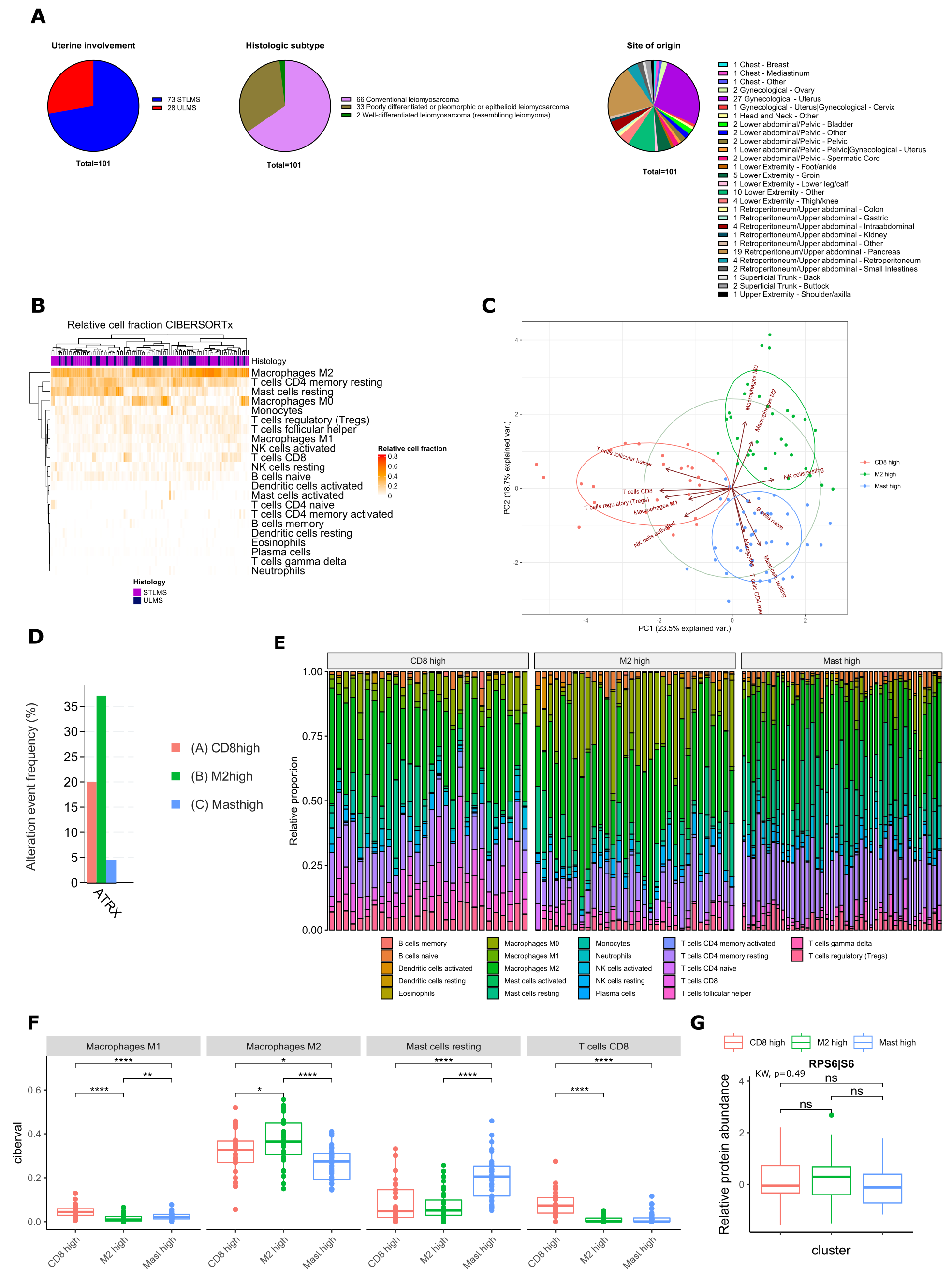

Supplement: Supplementary file 1 — Supporting Information [file CTM2-14-e1655-s005.tiff]

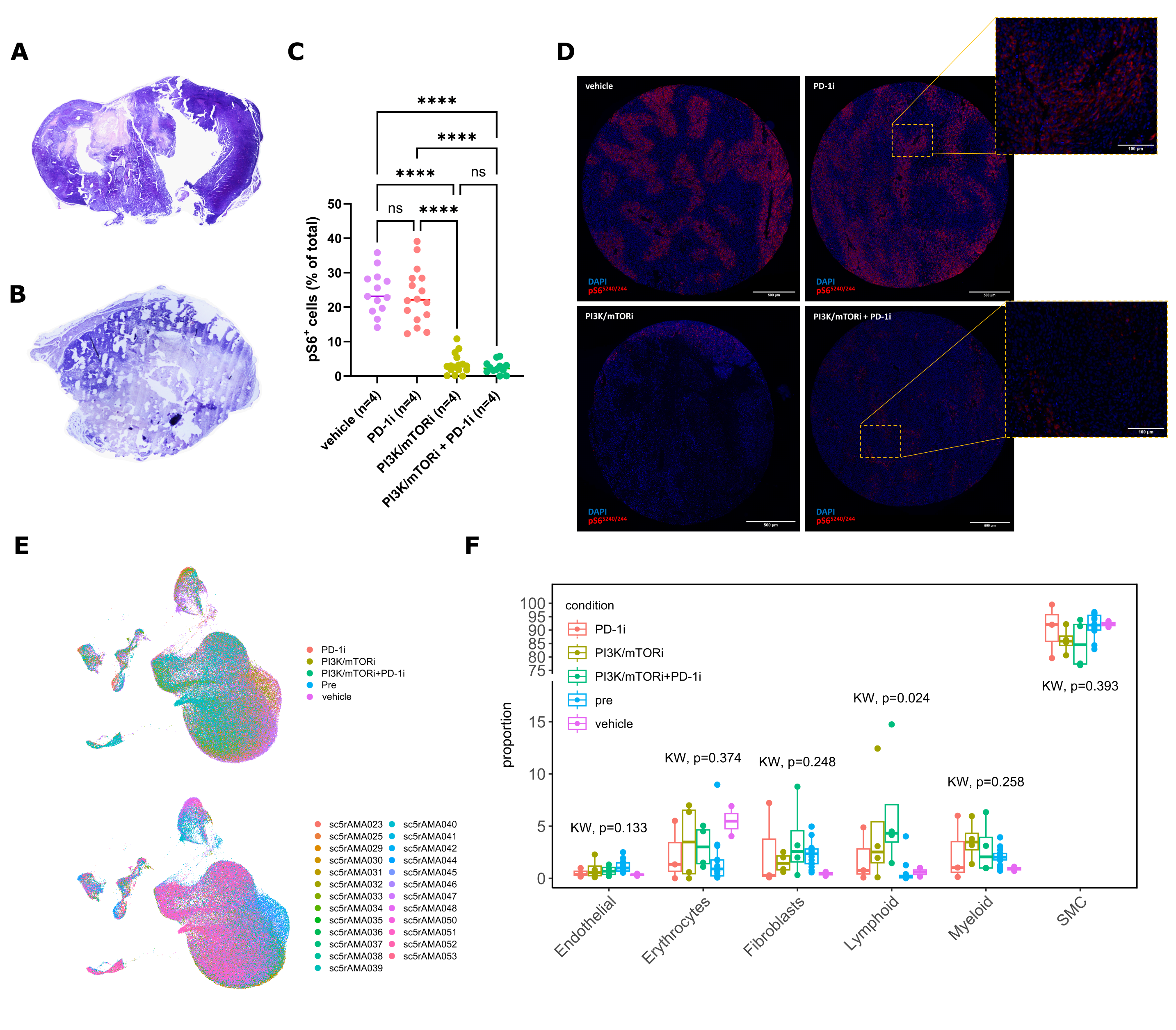

Supplement: Supplementary file 2 — Supporting Information [file CTM2-14-e1655-s003.tiff]

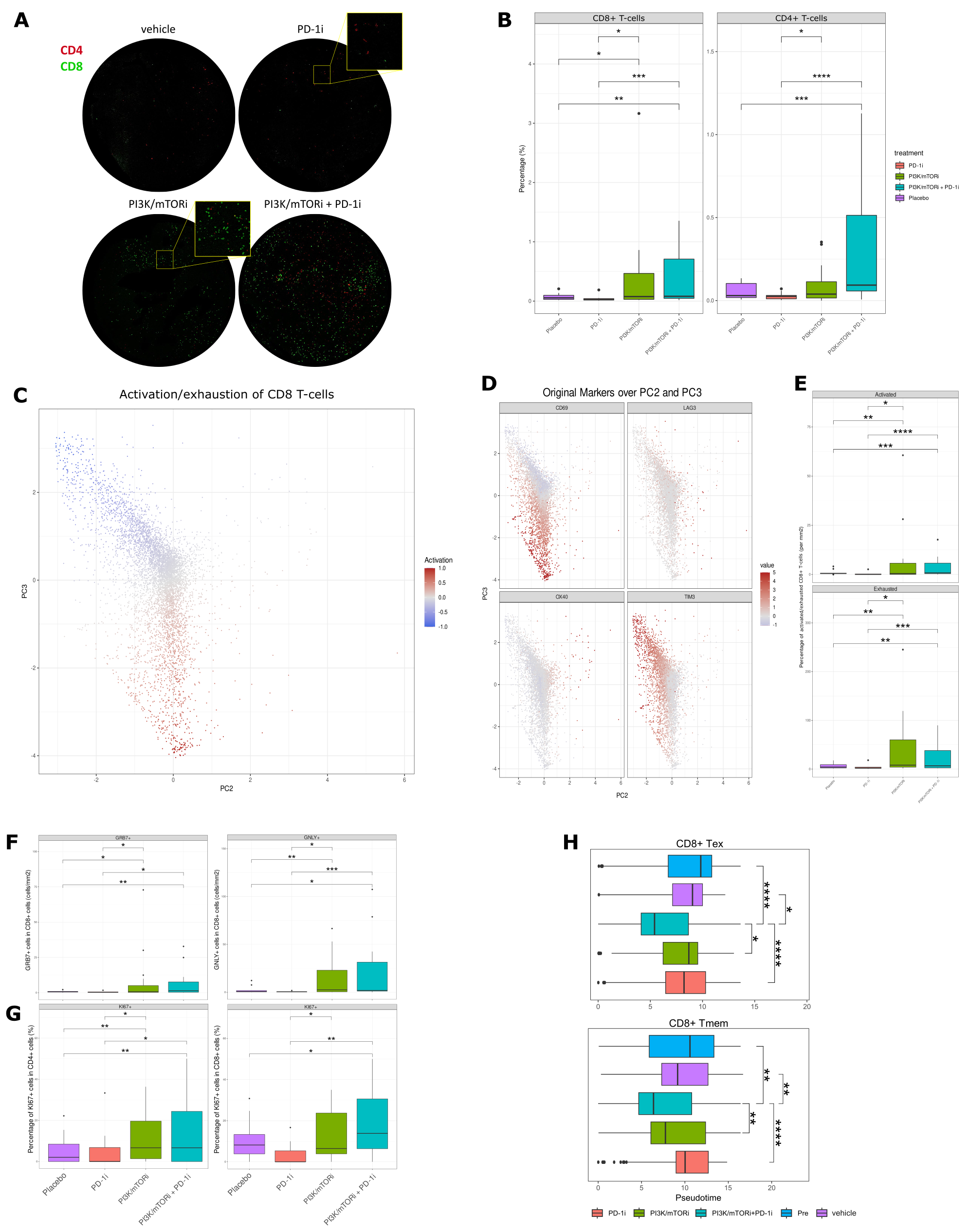

Supplement: Supplementary file 3 — Supporting Information [file CTM2-14-e1655-s006.tiff]

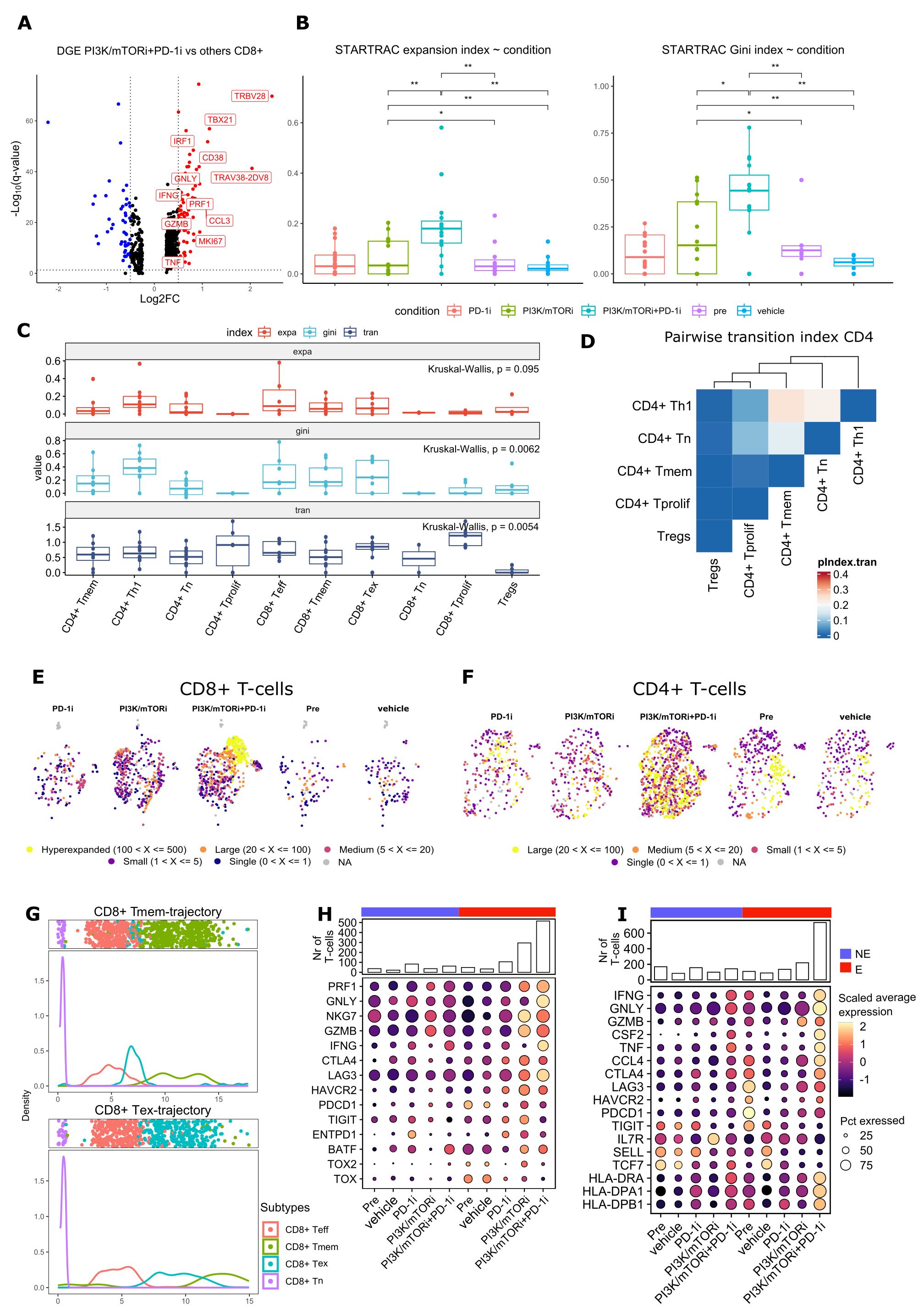

Supplement: Supplementary file 4 — Supporting Information [file CTM2-14-e1655-s004.tiff]

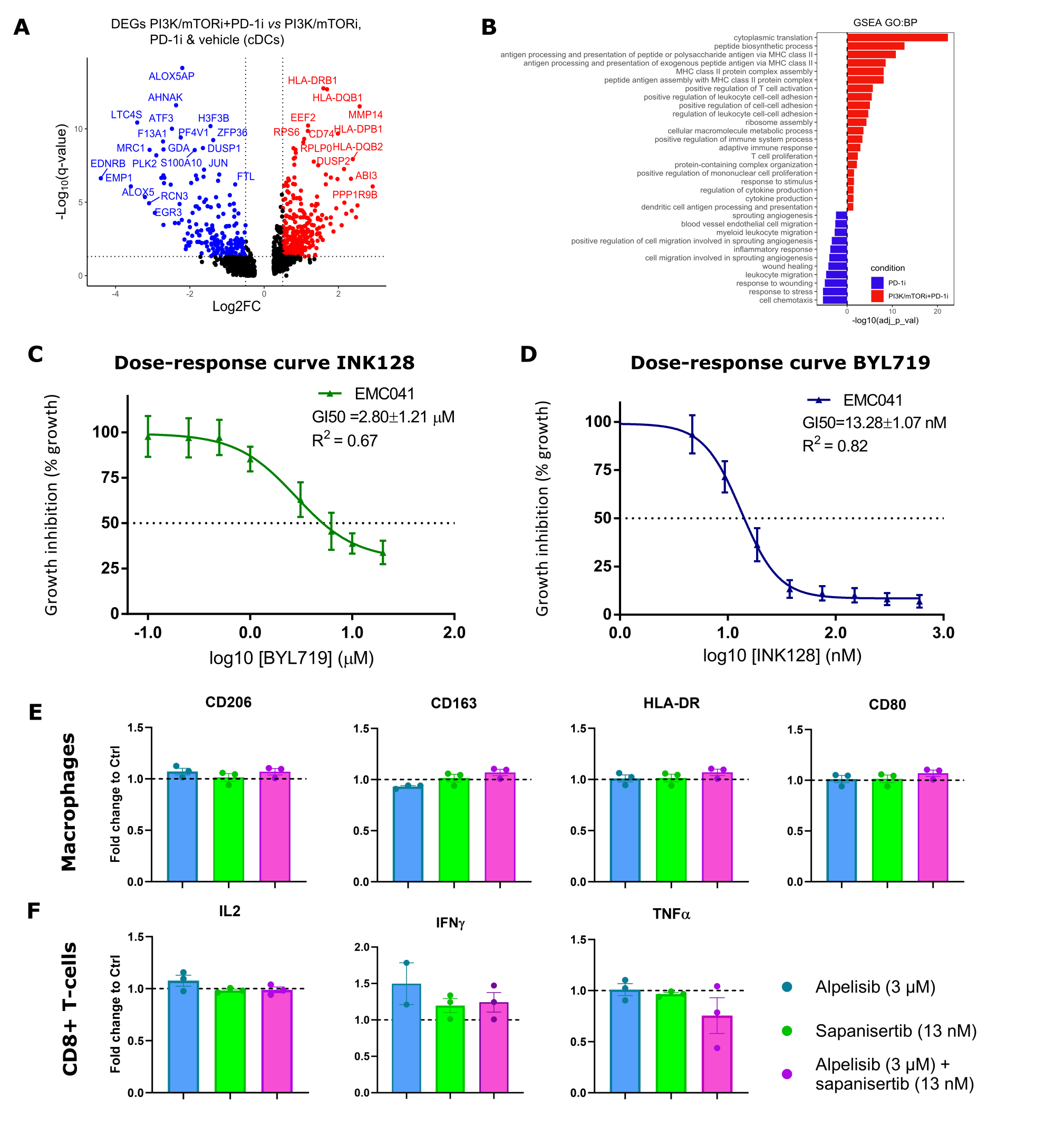

Supplement: Supplementary file 5 — Supporting Information [file CTM2-14-e1655-s001.tiff]

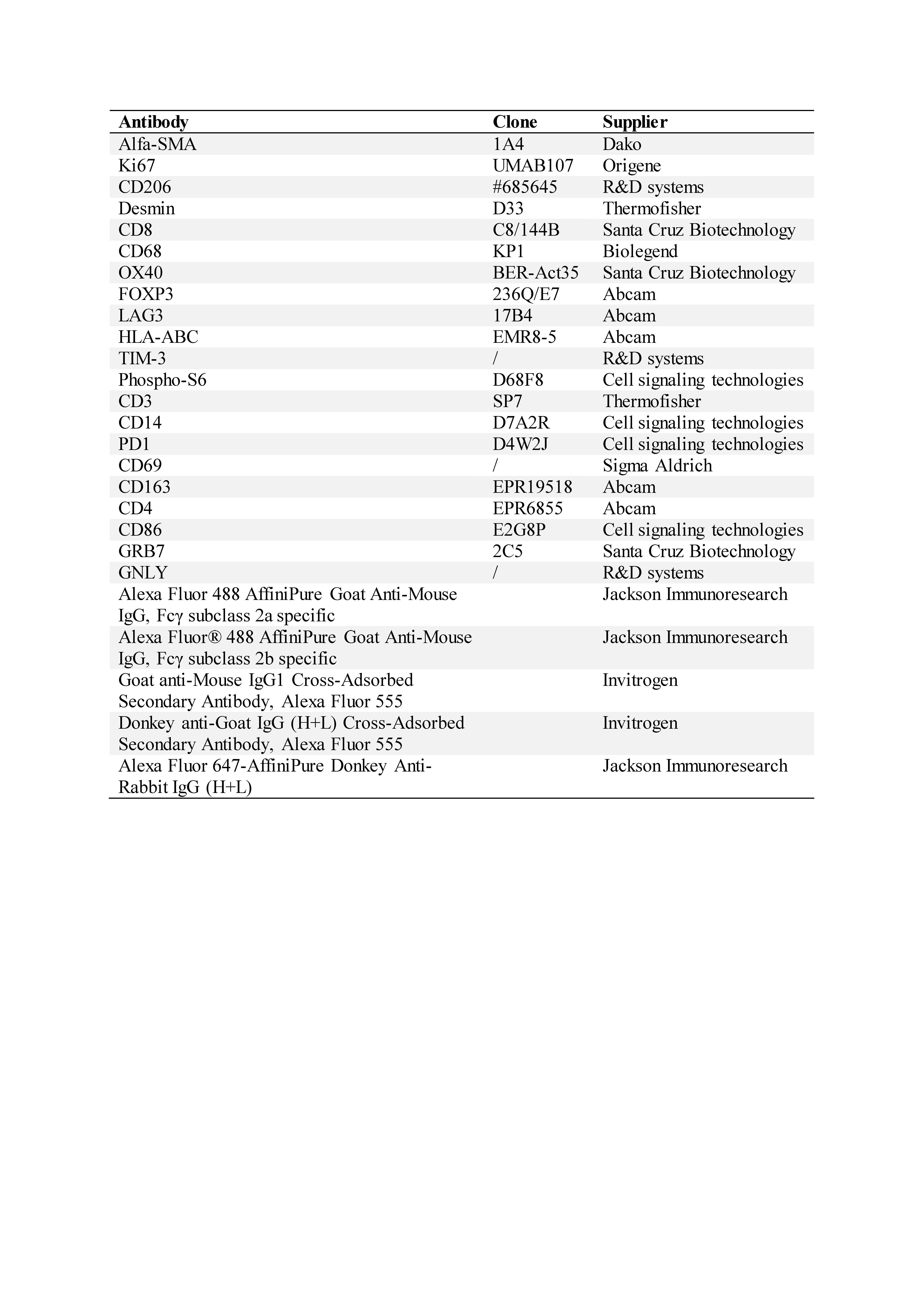

Supplement: Supplementary file 6 — Supporting Information [file CTM2-14-e1655-s002.jpg]
